# Dynorphin / kappa-opioid receptor regulation of excitation-inhibition balance toggles afferent control of prefrontal cortical circuits in a pathway-specific manner

**DOI:** 10.1038/s41380-023-02226-5

**Published:** 2023-08-29

**Authors:** Hector E. Yarur, Sanne M. Casello, Valerie S. Tsai, Juan Enriquez-Traba, Rufina Kore, Huikun Wang, Miguel Arenivar, Hugo A. Tejeda

**Affiliations:** 1 Unit on Neuromodulation and Synaptic Integration, National Institute of Mental Health, National Institutes of Health, Bethesda, MD USA; 2 NIH Graduate Partnership Program, Washington, DC USA

**Keywords:** Neuroscience, Physiology

## Abstract

The medial prefrontal cortex (mPFC) controls behavior via connections with limbic excitatory afferents that engage various inhibitory motifs to shape mPFC circuit function. The dynorphin (Dyn) / kappa-opioid receptor (KOR) system is highly enriched in the mPFC, and its dysregulation is implicated in neuropsychiatric disorders. However, it is unclear how the Dyn / KOR system modulates excitatory and inhibitory circuits that are integral for mPFC information processing and behavioral control. Here, we provide a circuit-based framework wherein mPFC Dyn / KOR signaling regulates excitation-inhibition balance by toggling which afferents drive mPFC neurons. Dyn / KOR regulation of afferent inputs is pathway-specific. Dyn acting on presynaptic KORs inhibits glutamate release from afferent inputs to the mPFC, including the basolateral amygdala (BLA), paraventricular nucleus of the thalamus, and contralateral cortex. The majority of excitatory synapses to mPFC neurons, including those from the ventral hippocampus (VH), do not express presynaptic KOR, rendering them insensitive to Dyn / KOR modulation. Dyn / KOR signaling also suppresses afferent-driven recruitment of specific inhibitory sub-networks, providing a basis for Dyn to disinhibit mPFC circuits. Specifically, Dyn / KOR signaling preferentially suppresses SST interneuron- relative to PV interneuron-mediated inhibition. Selective KOR action on afferents or within mPFC microcircuits gates how distinct limbic inputs drive spiking in mPFC neurons. Presynaptic Dyn / KOR signaling decreases KOR-positive input-driven (e.g. BLA) spiking of mPFC neurons. In contrast, KOR-negative input recruitment of mPFC neurons is enhanced by Dyn / KOR signaling via suppression of mPFC inhibitory microcircuits. Thus, by acting on distinct circuit elements, Dyn / KOR signaling shifts KOR-positive and negative afferent control of mPFC circuits, providing mechanistic insights into the role of neuropeptides in shaping mPFC function. Together, these findings highlight the utility of targeting the mPFC Dyn / KOR system as a means to treat neuropsychiatric disorders characterized by dysregulation in mPFC integration of long-range afferents with local inhibitory microcircuits.

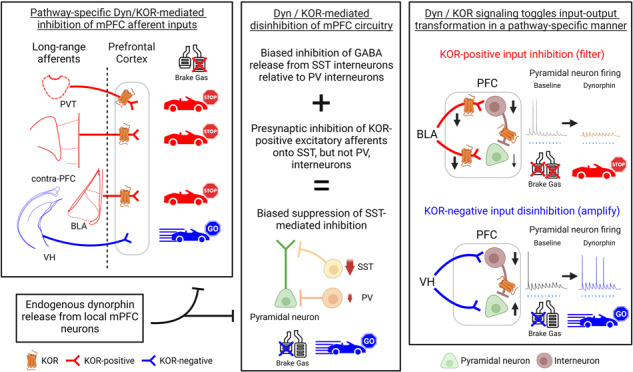

## Introduction

Cortical areas, including the medial prefrontal cortex (mPFC), coordinate excitatory inputs with local circuit inhibition to control downstream cortical and sub-cortical targets. The ability to switch how excitatory inputs control local circuits is essential for the mPFC to regulate a plethora of higher-order behaviors including decision-making in response to threats, affection, motivation, and goal-directed behavior [1–4]. Recent advances have been made in understanding the local microcircuitry and connections with limbic regions that are essential for the many facets of the mPFC to regulate behavior. However, our understanding of how mPFC and cortical circuits function has been largely based on how fast synaptic glutamatergic and GABAergic transmission shapes inter-cellular communication, despite the fact that the mPFC is enriched in neuropeptides and their cognate receptors [5–12]. Specifically, neuropeptides have been exploited as markers of specific cortical cell types and neuropeptide genes have provided experimental means to gain genetic access to sub-populations of cortical neurons. However, there is a paucity in studies that have examined the role of neuropeptides in modulating mPFC circuit function, including how mPFC circuits integrate information from long-range afferent inputs with local computations carried within mPFC excitatory and inhibitory local-circuit motifs.

Kappa-opioid receptors (KORs) and their endogenous ligand dynorphin (Dyn), are expressed in mPFC circuits [10–13]. Dyn release in the mPFC has been implicated in promoting negative affect and anxiety-like behavior, together with stress-induced increases in cardiovascular function [14–19]. Further, KOR phosphorylation (an index of recent KOR activation) has been observed in the mPFC after various stressors [16]. Collectively, these results suggest that Dyn / KOR signaling in mPFC circuits may help the mPFC regulate affective behaviors and adaptations to stress. KOR activation inhibits mPFC dopamine (DA) release and glutamatergic excitatory postsynaptic potentials (EPSPs) originating from the amygdala, but not hippocampal, inputs to the mPFC [14, 15]. These observations suggest that Dyn / KOR system recruitment may serve to modify how mPFC circuits incorporate excitatory drive from distinct afferent inputs, but this possibility has not been directly tested. Interestingly, KOR activation inhibits elevations in extracellular GABA driven by glutamate reuptake blockade in the mPFC, without modifying basal GABA levels [14]. This raises the possibility that KORs regulate recruitment of inhibitory networks by excitatory synaptic inputs. Thus, Dyn / KOR signaling may likely regulate mPFC function in a more complex manner than simply inhibiting afferent inputs to the mPFC. However, it is unclear how the Dyn / KOR system integrates itself into limbic-cortical circuits and impacts excitatory and inhibitory circuit motifs that regulate information flow into and out of the mPFC. Specifically, it is unclear (1) whether the Dyn / KOR system homogeneously regulates all afferent inputs to the mPFC, (2) how it may influence afferent-driven recruitment of mPFC inhibitory sub-networks, and (3) how this system shapes the way the mPFC integrates excitatory transmission from afferent inputs with polysynaptic inhibition to gate spike outputs.

Here, we address these gaps using a combination of whole-cell slice electrophysiology, viral and genetic approaches, optogenetics, and two-photon calcium imaging. In this study, we reveal that the expression of functional KORs, or lack thereof, in afferent inputs fundamentally changes the impact of Dyn / KOR signaling on input-output transformations in mPFC pyramidal neurons. This study advances our understanding of the neuropeptidergic motifs that shape mPFC information processing.

## Materials and methods

Detailed explanations of materials and methods are provided in the supplemental materials and methods section.

### Animals

Adult (>postnatal day 60) male and female C57/Bl6J WT, prodynorphin-Cre (PDyn-Cre), Somatostatin-Cre (SST-Cre), parvalbumin-Cre (PV-Cre), kappa opioid receptor-Cre (KOR-Cre), (KOR-loxP) mice were used. Mice were obtained from Jackson Laboratories. All procedures were approved by the National Institute of Mental Health Animal Care and Use Committee.

### Viral injections

Microinjections of different virus were targeted to mPFC, VH, PVT and BLA. Experiments were then performed approximately 4–8 weeks after injection.

### Retrobead retrograde tracing

Green XI retrobeads (Lumafluor) were microinjected (150–300 nl) into the mPFC in C57/Bl6J WT mice. Brains were removed for RNAscope in-situ hybridization 6-7 days after surgery.

### Ex-vivo whole cell electrophysiology

Whole-cell patch-clamp electrophysiology studies were performed as previously described [20, 21]. Cells were visualized using IR-DIC optics on an inverted Olympus BX5iWI microscope. For recordings, the recording chamber was perfused with a pump (World Precision Instruments) at a flow rate of 1.5–2.0 ml per minute with artificial cerebrospinal fluid (aCSF) containing 1.2 mM MgCl_2_ and 2.4 mM CaCl_2_. For biophysically isolated oEPSCs and oIPSCs cells were held at −55mV and +10 mV respectively. Monosynaptic oEPSCs and oIPSCs evoked by KOR^+^ cell stimulation were isolated with TTX (1 µM) and 4-AP (50 µM). For whole-cell recordings of intrinsic excitability, we utilized glass microelectrodes (3–5 MΩ) with K^+^-based internal solution. For oEPSCs and oIPSCs, excitation/inhibition balance, Rubi-GABA (5 µM) evoked IPSCs, MNI-glutamate (50 µM) evoked EPSC and MNI-glutamate evoked IPSCs at −55mV and +10 mV, respectively, we utilized glass microelectrodes (3–5 MΩ) with Cs^+^-based internal solution.

### RNAscope in-situ hybridization (ISH)

RNAscope ISH was conducted for kappa opioid receptor (*Oprk1*), parvalbumin (*Pvalb*), Somatostatin (*Sst*), VGLUT1 (*Slc17a7*), VGAT (*Slc32a1*), Prodynorphin (Pdyn) according using the RNAscope Fluorescent Multiplex Assay (Advanced Cell Diagnostics (ACD); Newark, CA, USA) as previously reported [14, 20]. Finally, slides were imaged on a confocal microscope (Olympus) with a 20x objective.

### Two-photon imaging

Slice calcium recordings were done using an upright FVMPE-RS multiphoton microscope (Olympus) with a 40X objective. Ca^2+^ signals were measured at 15 Hz using a 512 × 512 resonant scanner. Ca^2+^ signals were quantified by averaging peri-stimulation full-field fluorescence during baseline and after Dyn application.

## Results

### Dyn / KOR pathway-specific regulation of limbic excitatory inputs to the mPFC

**Fig. 1:**
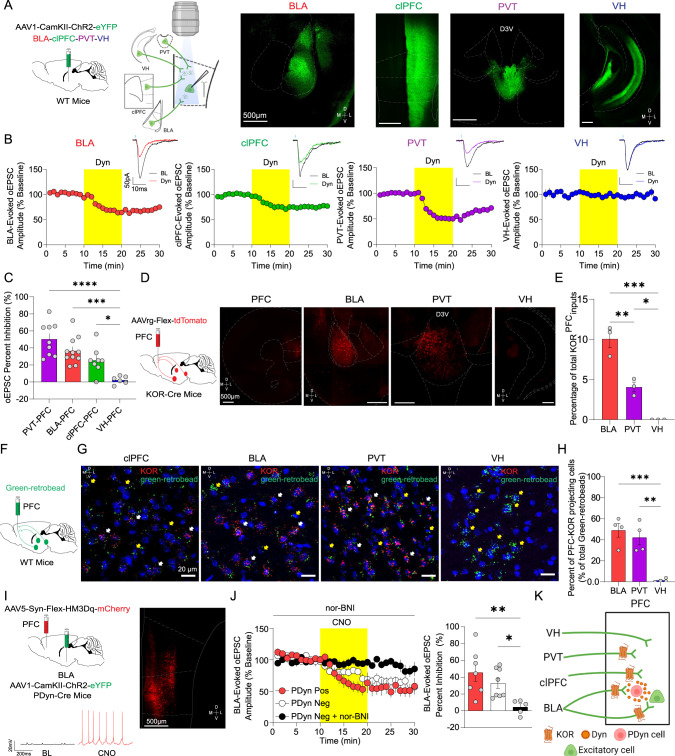
Pathway-specific regulation of mPFC excitatory afferents by the Dyn / KOR system. **A** Schematic diagram of the experimental design and representative image AAV1-CaMKII-ChR2-eYFP expression in the PVT, BLA, contralateral PFC, and VH. **B** Time course of the effect of KOR activation with Dyn (1 µM) on oEPSC amplitude (expressed as a percentage of baseline) in the mPFC from animals expressing ChR2-eYFP in the PVT, BLA, clPFC, or VH. Representative oEPSCs recorded in PFC from PVT (purple traces), BLA (red traces), clPFC (green traces), and VH (blue traces) during baseline (dark traces) and after Dyn (color traces) are shown. Data in this graph and the rest of the manuscript are presented as the mean ± SEM. **C** Percent inhibition of oEPSC amplitude evoked from different mPFC inputs by Dyn. **D** KOR-Cre mice were injected unilaterally with AAVrg-FLEX-tdT into the mPFC. Representative images showing retrogradely-labeled tdTomato-expressing KOR-positive cells in different mPFC input areas (clPFC, BLA, VH, and PVT). **E** Percentage of total KOR-containing projecting cells between different mPFC inputs. **F** Wild-type mice were injected bilaterally with green-retro beads into the mPFC. **G** Representative images of KOR mRNA (red puncta), retro beads (green puncta), and DAPI (blue) in mPFC input areas (clPFC, BLA, PVT, and VH). **H** Quantification of colocalization of KOR mRNA and green-retro beads in PVT, BLA, and VH. I. AAV1-CaMKII-ChR2-eYFP expression in the BLA and AAV5-Syn-FLEX-HM3Dq-mCherry in the mPFC of PDyn-Cre mice. Representative traces of CNO-induced activation excitatory DREADD in a mPFC mCherry-positive PDyn cell. **J** Time course of the effect of CNO activation (1 µM) on BLA-evoked oEPSC amplitude in PDyn-positive and negative mPFC cells in the presence or absence of nor-BNI (1 µM). Percentage of oEPSC inhibition induced by CNO application and co-application with nor-BNI. K. Pathway-specific KOR modulation of excitatory synapses in the mPFC. KORs inhibit BLA, clPFC, but not VH, afferents onto mPFC neurons.

The basolateral amygdala (BLA), paraventricular nucleus of the thalamus (PVT), contralateral PFC (clPFC), and ventral hippocampus (VH) are major limbic inputs to the mPFC. To determine whether presynaptic Dyn signaling via KOR inhibits afferents to the mPFC in a pathway-specific manner, we injected WT mice with AAV-CaMKII-ChR2-eYFP into the BLA, PVT, clPFC or VH, and recorded optogenetically evoked excitatory postsynaptic currents (oEPSCs) onto layer V pyramidal neurons (Fig. 1A). Dyn (Dynorphin A 1–17) inhibited excitatory inputs selectively from the PVT, BLA, clPFC, but not the VH (Fig. 1B, C). Dyn inhibition of BLA-evoked EPSCs was reversed by subsequent application of nor-BNI, a KOR antagonist, ten minutes after Dyn wash out, indicating that KOR signaling did not induce plasticity in BLA-mPFC synapses (Fig. S1A). Consistent with a presynaptic site of action, Dyn failed to modify excitatory currents evoked by glutamate uncaging (Fig. S1B). We subsequently injected AAVrg-FLEX-tdTomato in the mPFC of KOR-Cre mice to determine whether differential KOR expression may underlie pathway-specific inhibition of EPSCs that we observed (Fig. 1D). Retrogradely-labeled tdTomato cells that projected to the mPFC were observed in the BLA, PVT, clPFC and several higher-order telencephalon and midbrain regions, including the insular cortex, clPFC, piriform cortex, claustrum, perirhinal cortex, periamygdaloid cortex, and ventral tegmental area (VTA; Fig. 1D, E; Fig. S1C). Importantly, the VH lacked tdTomato-labeled neurons, corroborating the observed lack of Dyn regulation of VH inputs to the mPFC. Further, KOR mRNA expression was absent in the VH formation of WT mice where outputs to the mPFC originate but was present in the PVT and BLA, corroborating the observed lack of KOR-positive VH afferents to the mPFC (Fig. S1D). Consistent with retrograde tracing in KOR-Cre mice, retrograde labeling of mPFC-projecting cells using retrobeads demonstrated that KOR mRNA is expressed in mPFC-projecting cells in the BLA, PVT, and clPFC, but not mPFC-projecting VH neurons (Fig. 1F–H). Importantly, Dyn application failed to modify the frequency and amplitude of spontaneous excitatory postsynaptic currents (sEPSCs) onto layer V pyramidal neurons (Fig. S1E). Together, with the lack of effect of Dyn on glutamate uncaging evoked EPSCs (Fig. S1B), these results demonstrate that KOR activation does not regulate glutamate receptor function post-synaptically. The mPFC is the most interconnected area of the brain, receiving inputs from almost every other cortical region and a wide array of sub-cortical regions [22, 23]. However, our retrograde tracing studies in KOR-Cre mice demonstrated limited inputs originating from cortical and sub-cortical areas. In combination with the lack of Dyn effect on sEPSC frequency, these results collectively suggest that the majority of excitatory synapses innervating layer V pyramidal neurons are not modulated by presynaptic KOR signaling.

The source of Dyn acting on presynaptic KORs on BLA, PVT, and clPFC inputs is unclear. Indeed, injection of PDyn-Cre mice with AAV-expressing Cre-dependent Synaptophysin-GFP-2A-tdTomato revealed that mPFC Dyn neurons have extensive local connections within the mPFC (Fig. S1E). To determine whether local Dyn release regulates afferent inputs to the mPFC, we injected an AAV-CaMKII-ChR2-eYFP into the BLA and a Cre-dependent HM3Dq-expressing virus into the mPFC of PDyn-Cre mice (Fig. 1I). Chemogenetic activation of mPFC Dyn cells produced robust firing that persisted during drug application (Fig. 1I). CNO bath application inhibited BLA-evoked oEPSCs onto HM3Dq-positive Dyn neurons and Dyn-negative counterparts (Fig. 1J). This effect was not present in cells pretreated with the KOR antagonist nor-BNI. These results suggest that Dyn acts as a negative feedback signal on KOR-positive inputs onto mPFC Dyn cells, and collectively demonstrate that mPFC Dyn cells can broadly influence excitatory afferent drive of mPFC neurons in a pathway-specific manner onto both Dyn positive and negative neurons (Fig. 1K).

### Dyn / KOR signaling inhibits afferent-driven polysynaptic inhibition in a pathway-independent manner

**Fig. 2:**
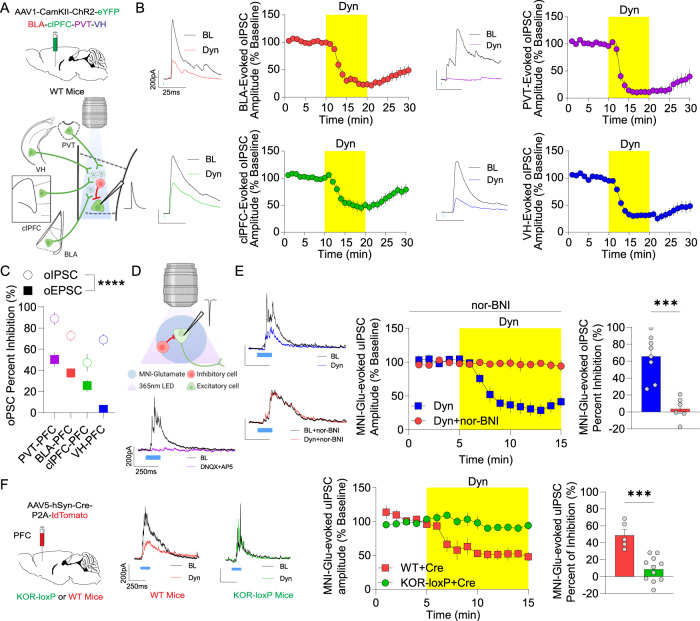
Dyn / KOR signaling inhibits afferent-driven polysynaptic inhibition in a pathway-independent manner. **A** Schematic diagram of the experimental design. AAV1-CaMKII-ChR2-eYFP expression in the PVT, BLA, contralateral PFC, and VH. **B** Time course of the effect of KOR activation with Dyn (1 µM) on the amplitude of oIPSC in the mPFC from animals expressing ChR2-eYFP in the PVT, BLA, clPFC, or VH. Representative oIPSCs recorded in the mPFC evoked by stimulation of PVT (purple traces), BLA (red traces), clPFC (green traces), and VH (blue traces) inputs during baseline (dark traces) and after the KOR agonist, Dyn (color traces). **C** Percentage inhibition of polysynaptic oIPSCs and direct oEPSCs by Dyn application between different mPFC inputs. **D** Schematic diagram and representative oIPSCs were evoked in mPFC principal neurons using MNI-Glutamate. Glutamate uncaging-evoked IPSCs are shown during baseline (dark traces) and after DNQX + AP5 (purple), Dyn (blue), or Dyn+nor-BNI (red). **E** Time course of the effect of KOR activation with Dyn on the amplitude (expressed as a percentage of baseline) of oIPSCs evoked by MNI-glutamate uncaging in the mPFC or co-application nor-BNI. Comparison of percent inhibition of oIPSCs evoked by MNI-glutamate by Dyn and in cells with concomitant Dyn and nor-BNI bath application. **F** KOR-loxP mice were injected bilaterally with AAV5-hSyn-Cre-P2A-tdTomato into the mPFC. Representative trace of oIPSCs evoked by MNI-glutamate uncaging. Traces of IPSCs during baseline (dark traces) and after Dyn in WT (red) or KOR-loxP (green) mice. Time course of the effect of KOR activation on the amplitude of oIPSCs evoked by MNI-glutamate in mPFC neurons from WT or KOR-loxP mice. Percent inhibition of oIPSC induced by MNI-glutamate by Dyn in WT and KOR-loxP mice.

Afferent inputs to the mPFC recruit polysynaptic inhibition to shape mPFC circuit function [5]. Since Dyn / KOR signaling inhibits the ability of glutamate reuptake inhibition to elevate extracellular GABA levels as assessed by microdialysis [14], it is possible that Dyn / KOR signaling may regulate polysynaptic inhibition in the mPFC recruited by afferent inputs. To test this, we injected AAV-expressing ChR2 into BLA, VH, PVT, or clPFC and recorded biophysically isolated optogenetically evoked polysynaptic IPSCs (opsIPSCs) onto layer V pyramidal neurons (Fig. 2A; Fig. S2A). opsIPSCs in mPFC neurons driven by afferent inputs from the BLA, VH, and thalamus are blocked by glutamate receptor antagonism (Fig. S2A; [24–26]). Unlike the pathway specific Dyn inhibition of EPSCs described above (Fig. 1), Dyn decreased polysynaptic inhibition in mPFC pyramidal neurons from all four afferents (Fig. 2B). This included polysynaptic inhibition driven by inputs from the VH, which do not express functional KORs (Fig. 1). Inhibition of opsIPSCs was more robust than inhibition of excitatory afferents (Fig. 2C). The selective KOR agonist Salvinorin A similarly inhibited afferent-driven polysynaptic inhibition (Fig. S2B). To test whether Dyn directly impacts GABA receptor function in pyramidal neurons, we measured inhibitory currents evoked by GABA uncaging and found that these currents were not modified by Dyn application (Fig. S2C) which indicates that KOR activation does not modify postsynaptic GABA receptor function. Furthermore, a KOR agonist failed to modify GABAergic miniature IPSC (mIPSCs) frequency (Fig. S2D), suggesting that KOR activation does not modify GABA release probability from a sufficiently large set of inhibitory synapses for us to detect an effect. In contrast, Dyn decreased spontaneous IPSC (sIPSC) frequency and sIPSC amplitude (Fig. S2E), suggesting that Dyn does not modify GABA transmission via a post-synaptic site of action but rather through effects on action potential-dependent activity within mPFC circuits. Taken together, these results suggest that Dyn suppresses excitation-driven recruitment of mPFC inhibitory circuits, and this may in part promote Dyn-mediated disinhibition.

### Dyn / KOR signaling within mPFC circuits contributes to Dyn-mediated disinhibition

Despite the lack of direct effects of Dyn on excitatory inputs from the VH to the mPFC, Dyn suppressed polysynaptic inhibition driven by the VH-mPFC pathway (Fig. 2C). This data indicates that the disinhibitory effect of Dyn might be mediated via Dyn/KOR signaling within local mPFC circuits. To test whether Dyn regulates recruitment of polysynaptic inhibition via actions on local circuitry, we uncaged glutamate to bypass excitatory inputs to the mPFC and examined opsIPSCs, which were completely blocked by the AMPA and NMDA receptor antagonists DNQX and AP5, respectively (Fig. 2D). Glutamate uncaging-evoked opsIPSCs were robustly inhibited by Dyn, an effect that was blocked by the KOR antagonist nor-BNI (Fig. 2E). To determine whether expression of KORs by mPFC neurons mediates the disinhibitory effects of Dyn, we genetically ablated KOR-expression within mPFC circuits (Fig. 2F). Dyn failed to inhibit glutamate uncaging-evoked opsIPSCs in KOR loxP mice injected with intra-mPFC AAV-Cre, an effect that was intact in Cre-expressing WT controls (Fig. 2F). This demonstrates that Dyn / KOR signaling contributes to disinhibition of mPFC circuits and provide a functional verification of successful KOR genetic ablation. Collectively, these results suggest that Dyn disinhibits mPFC pyramidal neurons by activating KORs embedded within mPFC circuits.

### mPFC KOR-expressing excitatory neurons engage local circuit inhibition

**Fig. 3:**
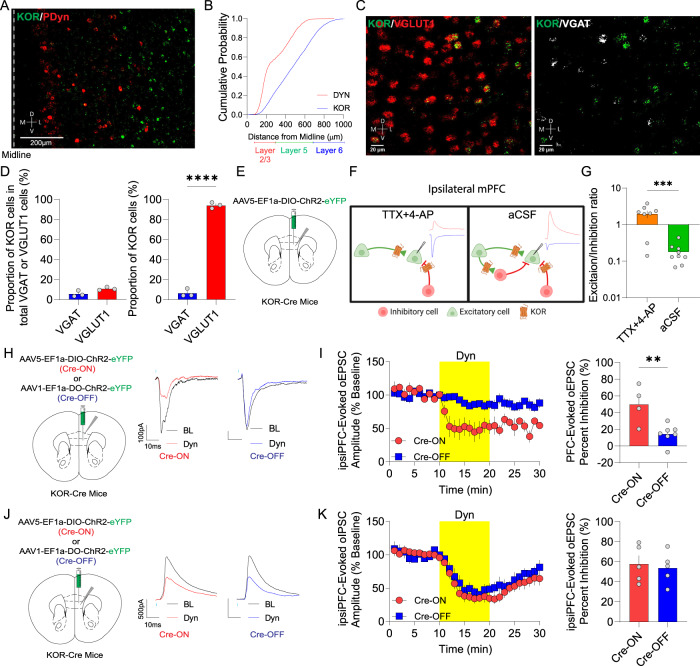
Dyn inhibits mPFC KOR-expressing neuron engagement of feedback inhibition. **A** Representative image of KOR mRNA (green puncta) and PDyn mRNA (red puncta) in the mPFC of a WT mouse. **B** Cumulative probability of KOR and PDyn mRNA across PFC layers. **C** Representative image of KOR mRNA (green puncta) and VGLUT1 mRNA (red puncta) and VGAT mRNA (white puncta) in the mPFC of a WT mouse. **D** Mean percentage of KOR mRNA-positive cells expressing in total VGAT and VGLUT1 mRNA. Proportion of total KOR mRNA-positive cells that co-express VGAT and VGLUT1 mRNA. **E** KOR-Cre mice were injected unilaterally with AAV5-EF1α-DIO-ChR2-eYFP into the mPFC. **F** Schematic of electrophysiological analysis of monosynaptic connectivity by mPFC KOR-positive neurons or polysynaptic recruitment of inhibition. Representative traces of oEPSC (blue) and oIPSC (red) evoked by optogenetic stimulation of mPFC KOR-containing cells. **G** Comparison of excitation/inhibition ratios evoked by mPFC KOR cell stimulation in the presence of TTX + 4-AP or aCSF. **H** Schematic showing that KOR-Cre mice were injected unilaterally with AAV5-EF1α-DIO-ChR2-eYFP (Cre-ON) or AAV1-EF1α-FLOX-ChR2-eYFP (Cre-OFF) into the mPFC. Representative oEPSCs traces from mPFC neurons of animals injected with Cre-ON or Cre-OFF into the mPFC during baseline (dark traces) and after Dyn (color traces) are shown. **I** Time course of the effect of Dyn (1 µM) on oEPSC amplitude of mPFC KOR cell evoked oEPSC from animals expressing in KOR-containing (Cre-ON) or KOR-lacking (Cre-OFF) ChR2-eYFP in the ipsilateral mPFC. Percentage of oEPSC inhibition by Dyn application between KOR-containing (Cre-ON) and KOR-lacking (Cre-OFF) ChR2-eYFP in the ipsilateral PFC. **J** KOR-cre mice were injected with AAV5-EF1α-DIO-ChR2-eYFP (Cre-ON) or AAV1-EF1α-FLOX-ChR2-eYFP (Cre-OFF) into the mPFC. Representative oIPSCs traces from mPFC neurons from animals injected with Cre-ON or Cre-OFF ChR2 into the mPFC during baseline (dark traces) and after Dyn (color traces) are shown. **K** Time course of the effect of Dyn on the amplitude of oIPSCs in mPFC pyramidal neurons from animals expressing Cre-ON ChR2 in KOR-containing or Cre-OFF ChR2 in KOR-lacking in the ipsilateral mPFC. Percentage of oIPSC inhibition by Dyn application between KOR-containing (Cre-ON) and KOR-lacking (Cre-OFF) ChR2-eYFP in the ipsilateral PFC.

We next sought to dissect the impact of Dyn / KOR signaling on intra-mPFC inhibitory motifs (Fig. 2). If Dyn / KOR signaling within mPFC circuits promotes disinhibition, then KOR-expressing neurons are predicted to be embedded within mPFC circuits. One possibility is that Dyn / KOR signaling acts as an intercellular communication motif via differential expression of Dyn and KOR across mPFC cell types. An alternative, but not mutually exclusive, possibility is that the Dyn / KOR system functions as an autocrine signal in cells co-expressing Dyn and KOR. Contrary to the latter hypothesis, we showed that Pdyn and KOR mRNA-expression did not overlap within mPFC circuits (Fig. 3A). Specifically, Pdyn mRNA-positive neurons were localized in more superficial layers than KOR mRNA positive neurons (Fig. 3B). Further, KOR mRNA was primarily expressed by excitatory neurons of the mPFC, with sparser labeling in VGAT-expressing neurons (Fig. 3C, D). These results suggest that Dyn / KOR signaling may act as a conduit for selective interactions between distinct neurons within a mPFC microcircuit. Opposite to Dyn, KOR-positive excitatory and inhibitory neurons were localized in deeper layers than KOR-negative counterparts in both the dmPFC (prelimbic; Fig. S3A) and vmPFC (infralimbic; Fig. S3B), demonstrating that KOR-containing excitatory neurons represent a sub-population of mPFC principal neurons and interneurons.

Given that Dyn has potent disinhibitory effects via actions on mPFC microcircuits, the observation that KOR was primarily expressed in excitatory neurons was unexpected. Thus, we hypothesized that KOR-positive excitatory neurons recruit inhibitory mPFC components and that Dyn decreases feedback inhibition carried by KOR-positive neurons. We observed that KOR-positive mPFC neurons established putative synaptic connections throughout mPFC layers using KOR-Cre mice injected with AAV-expressing Cre-dependent Synaptophysin-GFP-2A-tdTomato (Fig. S3C). To identify whether excitatory and inhibitory KOR-positive cells established functional monosynaptic connections within local ipsilateral mPFC (ipsiPFC) circuits, we recorded biophysically-isolated oEPSCs and oIPSCs in the presence of TTX and 4-AP from ipsiPFC neurons. Optogenetic stimulation of KOR-positive cells in KOR-Cre mice injected with Cre-dependent ChR2 in the ipsilateral mPFC (Fig. 3E, F; Fig. S3D, E) produced monosynaptic oEPSCs in the majority of neurons recorded, while only half received both direct oEPSCs and oIPSCs with similar onset latencies (Fig. S3E, F). From cells that received both excitatory and inhibitory connections, excitatory responses were stronger (Fig. 3G), consistent with preferential expression of KOR mRNA in excitatory neurons. Considering KOR mRNA is mostly expressed in excitatory neurons, we determined whether mPFC KOR-positive neurons engage local circuit inhibition. We recorded oEPSCs/oIPSCs evoked by optogenetic stimulation of KOR-positive neurons in the ipsilateral mPFC in the absence of TTX/4-AP to permit the recruitment of inhibitory networks within the mPFC. In contrast to monosynaptic responses evoked from KOR-expressing neurons, oIPSCs evoked in aCSF lacking TTX/4-AP had significantly longer onset latencies and were significantly larger in amplitude than oEPSCs (Fig. 3G, Fig. S3E), demonstrating that excitatory KOR-expressing neurons potently recruit feedback inhibition within mPFC microcircuits. Collectively, these results demonstrate that KOR-expressing neurons in the mPFC are localized in mPFC deeper layers, and robustly engage local circuit feedback inhibition, providing one potential substrate by which Dyn / KOR signaling disinhibits mPFC circuits.

Expression of KOR mRNA in excitatory mPFC cells implies that Dyn may act on these cells to (1) suppress intrinsic excitability and/or (2) presynaptically inhibit neurotransmitter release from excitatory mPFC KOR cells within the local circuit. Contrary to the first hypothesis, Dyn bath application failed to evoke direct hyperpolarizing currents in KOR-expressing mPFC neurons (Fig. S3G). However, all KOR-expressing cells responded with inhibitory hyperpolarizing currents in response to GABA_B_ receptor activation with baclofen (Fig. S3H). This suggests KORs do not likely couple to GIRKs or other somatodendritic voltage-gated ion channels in mPFC pyramidal cells that regulate intrinsic excitability under these conditions, though these cells have the capacity to be directly hyperpolarized by Gi-coupled GPCRs. It is possible that KOR activation may inhibit glutamate release within ipsilateral mPFC microcircuits, similar to what was observed with clPFC connections (Fig. 1). To test this, we selectively expressed ChR2 in KOR-positive cells (Cre-ON) and recorded oEPSCs in pyramidal neurons in the ipsiPFC (Fig. 3H). Dyn bath application inhibited oEPSCs in ChR2-negative mPFC neurons (Fig. 3I). This effect was still present when monosynaptic currents were isolated using TTX/4-AP (Fig. S3I), indicating that Dyn inhibition is through a direct action on KOR-positive neurons and does not inhibit glutamate release via actions on voltage-gated sodium channels or 4-AP-sensitive potassium channels. Moreover, oEPSCs evoked from KOR-negative neurons (Cre-OFF approach) were unaffected by Dyn application. These results suggest that mPFC KORs inhibit local circuit excitatory connections established by KOR-expressing pyramidal neurons but do not directly hyperpolarize pyramidal neurons. In addition to regulating local excitatory collaterals, it is possible that functional KOR may be expressed in terminals from KOR-positive projection neurons in output regions, as exemplified by Dyn suppression of clPFC afferents (Fig. 1).

We subsequently determined whether inhibition driven by activation of KOR-positive and negative neurons was regulated by Dyn in KOR-Cre mice injected with Cre-ON and Cre-OFF ChR2, respectively, in the mPFC. In mice expressing Cre-ON ChR2, oIPSCs evoked by optogenetic activation of KOR-expressing neurons were robustly inhibited by Dyn (Fig. 3J, K). Since oIPSCs evoked under these conditions were primarily mediated by polysynaptic inhibition and not monosynaptic GABA release (Fig. 3G), one possible interpretation is that Dyn may be suppressing KOR-positive excitatory synapses that recruit inhibitory microcircuits. Not mutually exclusive, Dyn may also be inhibiting monosynaptic oIPSCs recruited by KOR-positive neurons that make up a smaller fraction of the total oIPSC. Surprisingly, oIPSCs evoked by KOR-negative cells were similarly inhibited by Dyn (Fig. 3J, K). However, since KOR-negative cells engage inhibitory circuits that are suppressed by Dyn, these results suggest that KOR-positive mPFC neurons, such as interneurons, downstream of KOR-negative excitatory cells contribute to Dyn-mediated disinhibition.

### Dyn / KOR signaling directly inhibits GABAergic transmission from sub-populations of mPFC interneurons

**Fig. 4:**
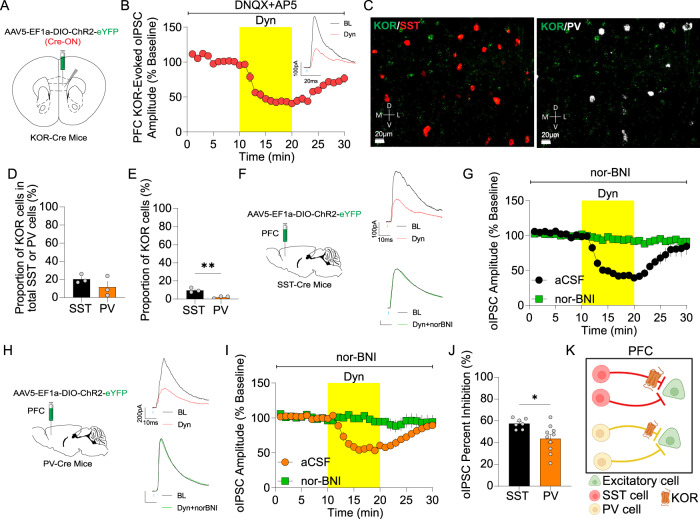
Dyn / KOR signaling directly inhibits GABAergic transmission from sub-populations of mPFC interneurons. **A** Schematic showing that KOR-Cre mice were injected unilaterally with AAV5-EF1α-DIO-ChR2-eYFP (Cre-ON) into the mPFC. **B** Time course of the effect of Dyn on the amplitude of oIPSCs in the presence of DNQX + AP5 in mPFC pyramidal neurons from animals expressing Cre-ON ChR2 in KOR-containing in the ipsilateral mPFC. Representative oIPSCs traces from mPFC neurons from animals injected with Cre-ON ChR2 into the mPFC during baseline (dark traces) and after Dyn (red traces) are shown. **C** Representative image of KOR mRNA (green puncta), SST mRNA (red puncta), and PV mRNA (white puncta) in the mPFC of a WT mouse. **D** Mean percentage of KOR mRNA-positive cells expressing in total SST and PV mRNA. **E** Proportion of total KOR mRNA-positive cells that co-express SST and PV mRNA. **F** SST-Cre mice were injected bilaterally with AAV5-EF1α-DIO-ChR2-eYFP PFC. Traces are shown during baseline (dark traces) and after Dyn (1 μM; red), and Dyn + nor-BNI (1 µM; green) are shown. **G** Time course of the effect of Dyn on the amplitude of oIPSCs in principal neurons from animals expressing ChR2 in SST-positive interneurons in the presence or absence of nor-BNI. H. Schematic depicting the injection of AAV5-EF1α-DIO-ChR2-eYFP into the mPFC of PV-Cre mice. Traces during baseline (dark traces) and after Dyn (red), and Dyn+nor-BNI (green) are shown. **I** Dyn inhibits the amplitude of PV-evoked oIPSC on mPFC pyramidal neurons in the presence or absence of nor-BNI. **J** Percentage of oIPSC inhibition by Dyn between PV or SST evoked by ChR2-eYFP in the mPFC. **K** Model representing that KOR inhibits oIPSCs evoked from SST- and PV-positive interneurons in the mPFC.

Since KOR expression was observed in a subset of inhibitory interneurons (Fig. 3C, D), we tested the hypothesis that Dyn inhibits GABA release from KOR-expressing inhibitory neurons. To this end, we injected Cre-dependent ChR2-expressing virus in the mPFC of KOR-Cre mice to label KOR-positive cells and blocked glutamate receptors to isolate inhibitory synaptic transmission from KOR-positive neurons to mPFC ChR2-negative principal neurons (Fig. 4A). Dyn bath application inhibited evoked oIPSCs in ChR2-negative mPFC neurons (Fig. 4B). These results suggest Dyn acts on mPFC KOR expressed in inhibitory neurons to inhibit GABA release. Next, we sought to identify whether KOR mRNA was differentially expressed in PV- and SST-neurons, two major classes of interneurons in the mPFC that make up the majority of inhibitory cells. KOR mRNA expression was observed in both PV- and SST-subpopulations (Fig. 4C–E). The proportion of KOR mRNA was higher in SST- than PV-positive neurons (Fig. 4E), but the relative abundance of KOR mRNA was similar in PV- and SST-positive neurons, as well as putative excitatory KOR-positive neurons (Fig. S4A). PV and SST-immunoreactive neurons were also observed in KOR-Cre mice injected with Cre-dependent virus expressing tdTomato (Fig. S4B). PV-positive, SST-positive and KOR mRNA-positive neurons were all similarly distributed across mPFC layers (Fig. S4C). Further, in KOR-Cre mice we injected a Cre-dependent GFP virus under the control of the mDlx promoter, which primarily permits genetic access to PV- and SST-positive interneurons, to label KOR-positive mPFC interneurons. GFP-positive cells had fast-spiking and non-fast-spiking electrophysiological properties, consistent with expression of KOR in multiple sub-populations of mPFC interneurons (Fig. S4D, E). In contrast, labeling all KOR cells, and hence biasing towards excitatory mPFC KOR cells, resulted in labeled cells with homogenous regular spiking properties typical of subsets of excitatory principal neurons (Fig. S4F, G). Putative excitatory KOR-positive cells had different physiological properties than KOR-positive mPFC interneurons (Fig. S4H). These results are consistent with KOR-expression being limited to a subpopulation of excitatory and inhibitory cells. To explore whether KORs differentially inhibit GABA release from SST- and PV-interneurons, we injected SST-Cre and PV-Cre mice with AAV-DIO-ChR2-eYFP into the mPFC and recorded oIPSCs in pyramidal neurons (Fig. 4F, H). Dyn inhibited monosynaptic oIPSCs from SST- and PV-expressing neurons via a presynaptic site of action, an effect blocked by pretreatment with the KOR antagonist nor-BNI (Fig. 4G, I; Fig. S4I, J). The inhibitory effect of Dyn on SST-evoked oIPSCs was significantly larger than the effect on PV-evoked oIPSCs (Fig. 4J), consistent with KOR mRNA expression in a larger sub-population of SST-interneurons than PV interneurons. There is a general agreement that KOR inhibition is larger in SST neurons relative to PV neurons between electrophysiological and in-situ hybridization results. However, it must be acknowledged that the electrophysiology suggests that at least 50–60% of SST- and PV-expressing neurons/fibers are KOR-positive, while in-situ hybridization experiments suggest only around 10–20% of cells had detectable KOR mRNA transcripts. Several factors may contribute to these discrepancies. This includes but is not limited to, the following possibilities. (1) Non-linear relationships between mRNA expression and functional proteins. (2) Alternative splice variants of KOR mRNA have been documented and these have been shown to have differential subcellular compartmentalization in the soma vs. processes and be differentially expressed in distinct CNS regions and potentially cell types [27, 28]. This is of relevance given that KOR mRNA may be translated locally in axon terminals [29]. (3) It is also possible that there is preferential connectivity of KOR-positive interneurons within layer V where we focused the present study. Indeed, the distribution of KOR-positive inhibitory neurons was shifted to layer V relative to KOR-negative interneurons (Fig. S3A, B and Fig. S4A, B). (4) Lastly, it is also possible that there are potential changes in KOR expression and/or function induced by slicing procedures necessary for electrophysiological studies that in-situ hybridization approaches are not subject to. Thus, KORs may disinhibit mPFC circuits through suppression of both SST and PV interneuron-mediated inhibition, with a bias towards robust suppression of SST-mediated inhibition (Fig. 4K).

### Dyn / KOR signaling inhibits feedforward inhibition mediated by SST, but not PV, mPFC interneurons

**Fig. 5:**
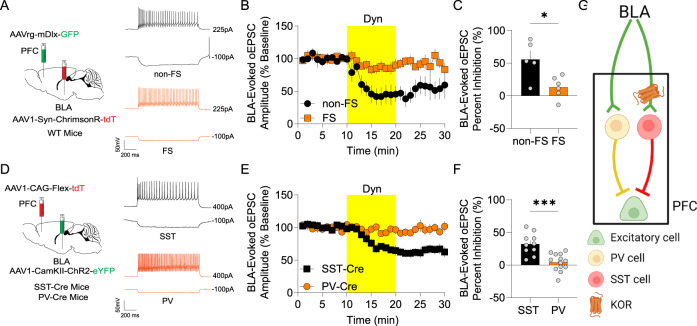
Dyn / KOR signaling inhibits feedforward inhibition by sub-networks of mPFC interneurons. **A** Schematic depicting WT mice injected with AAV1-Syn-ChrimsonR-tdT into the BLA and AAVrg-mDlx-GFP into the mPFC. Representative traces at different current steps of evoked action potentials in mPFC fast-spiking interneurons (FSIs; orange) and non-fast spiking interneurons (non-FSI; black). **B** Time course of the effect of Dyn on the amplitude of BLA-evoked oEPSCs in FSIs or non-fast spiking interneurons (non-FSI). **C** Dyn-induced inhibition of BLA-evoked oEPSCs (percent inhibition) in mPFC FSIs and non-FSIs. **D** Schematic depicting injection of AAV1-CaMKII-ChR2-eYFP into the BLA and AAVrg-Flex-tdT into the mPFC of SST-Cre or PV-Cre mice. Representative non-fast-spiking and fast-spiking properties from SST-positive (black) and PV-positive (orange) mPFC interneurons, respectively. **E** Time course of the effect of Dyn on BLA-evoked oEPSC amplitude in mPFC SST or PV. **F** Percentage of oEPSC inhibition by Dyn between PV or SST evoked by ChR2-eYFP positive terminals in the mPFC. **G** Model representation of parallel pathway-specific KOR modulation of BLA afferents to the mPFC. KOR inhibits BLA inputs onto mPFC non-FSI SST-positive, but not PV-positive FSIs.

Excitatory inputs to GABAergic interneurons by limbic afferents recruit rapid feedforward inhibition that is essential for appropriate mPFC function and control of behavior [5, 6]. To determine whether presynaptic KORs regulate excitatory inputs to distinct GABAergic neurons, we injected WT mice with AAV-hSyn-Chrimson-tdTomato into the BLA and AAVrg-mDlx-eGFP into the mPFC and evoked BLA-driven oEPSCs in Dlx-positive GABAergic interneurons (Fig. 5A [30]); Intrinsic firing properties of GFP-expressing neurons were determined, and cells were divided into fast-spiking and non-fast-spiking neurons (Fig. 5A). Interestingly, Dyn inhibited BLA oEPSCs onto non-fast-spiking mPFC Dlx-positive interneurons, but not Dlx-positive fast-spiking interneurons (Fig. 5B, C). These results suggest that BLA inputs innervating SST-interneurons may differentially express KORs. Given that non-fast-spiking interneurons may correspond to a plethora of inhibitory neurons, including SST interneurons, we further explored the role of Dyn in regulating inputs from the BLA onto SST- and PV-interneurons, which largely encompass various non-fast spiking and fast-spiking interneuron classes, respectively ([5–7, 31]; Fig. 5D). To this end, we injected SST-Cre and PV-Cre mice with AAV-expressing ChR2 into the BLA and AAV-FLEX-tdTomato into the mPFC of mice to specifically record oEPSCs onto SST and PV neurons, respectively (Fig. 5D). Dyn inhibited excitatory inputs from the BLA onto SST-expressing, but not PV, interneurons (Fig. 5E, F). Thus, in addition to inhibiting direct excitatory inputs to mPFC principal neurons from KOR-expressing inputs, the Dyn / KOR system may also shape feedforward inhibition coming into the mPFC by inhibiting excitatory synapses onto SST-positive interneurons, but not PV interneurons (Fig. 5G). Taken together, these results demonstrate that Dyn / KOR signaling disinhibits mPFC circuits via biased regulation of inhibitory subnetworks. Specifically, presynaptic KORs inhibit excitatory drive of SST-interneurons and suppress GABA release from subsets of inhibitory KOR-positive SST-interneurons, reducing SST-mediated feedforward inhibition via two independent mechanisms. In contrast, there is less Dyn / KOR regulation of PV interneuron-mediated feedforward inhibition as excitatory synapses innervating these neurons are not regulated by KORs and PV cell KOR mRNA-expression and Dyn suppression of PV neuron output is limited relative to SST-interneurons.

### Dyn / KOR signaling bidirectionally shapes input-output transformations in a pathway-dependent manner

**Fig. 6:**
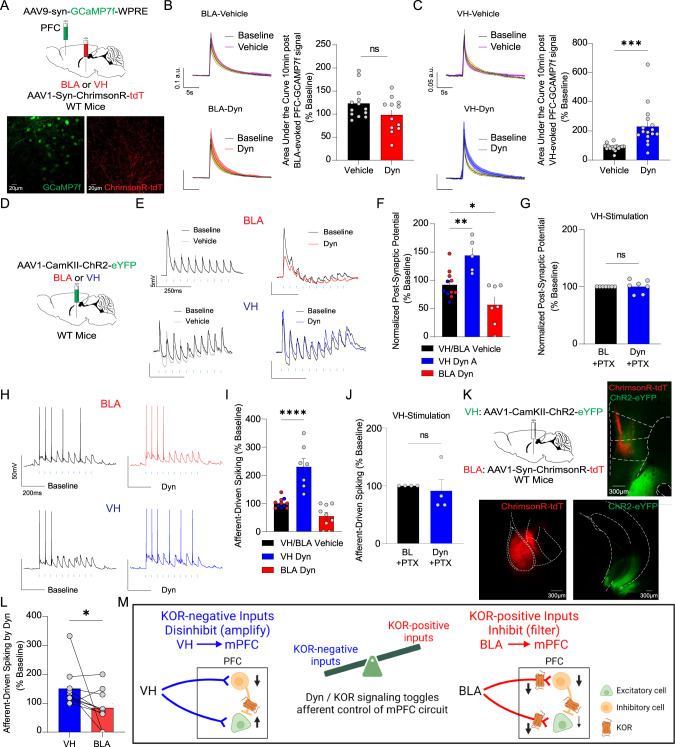
Dyn / KOR signaling bidirectionally shapes input-output transformations in a pathway-dependent manner. **A** WT mice were injected bilaterally with AAV1-Syn-ChrimsonR-tdT in the BLA or VH and AAV9-syn-GCaMP7f-WPRE in the PFC. Representative two-photon images of PFC GCaMP7f (green) and ChrimsonR (red) expression in PFC. **B** Left, representative traces of GCaMP7f signal for vehicle and Dyn bath application. Right, area under the curve of GCaMP7f signal evoked by a train stimulation of BLA inputs in mPFC slices. **C** Same as for B, but for VH inputs. **D** Experimental scheme wherein WT mice were injected bilaterally with AAV1-CaMKII-ChR2-eYFP in the BLA or VH. **E** Representative traces of PSPs in mPFC neurons in response to train stimulation of BLA inputs (10 pulses at 20 Hz), in the presence of vehicle (gray) or Dyn (1 µM); BLA (red); VH (blue). Blue lines indicate the timing of LED pulses. **F** The effect of Dyn / KOR signaling on the area under the curve of PSPs evoked by train stimulation of VH or BLA inputs normalized to baseline. **G** Picrotoxin on Dyn / KOR signaling on the area under the curve of PSPs evoked by train stimulation of VH inputs normalized to baseline. **H** Afferent-driving spikes mPFC neurons in response to train stimulation (10 pulses at 20 Hz) of BLA or VH inputs during baseline (black traces) and in the presence of Dyn (red or blue traces). Blue lines indicate the timing of LED pulses. **I** Mean number of spikes evoked by train stimulation of VH or BLA inputs in PFC after Dyn application relative to baseline. **J** Picrotoxin occludes Dyn enhancement of VH-driven spiking. **K** Mice were injected bilaterally with AAV1-CaMKII-ChR2-eYFP in the BLA and AAV1-Syn-ChrimsonR-tdT in the VH. **L** Number of spikes evoked by train stimulation of VH and BLA inputs in PFC in the same neuron in response to Dyn. **M** Dyn / KOR signaling filters KOR-positive and amplifies KOR-negative inputs, toggling how individual mPFC neurons are controlled by converging VH and BLA afferents.

Our previous results suggest that Dyn / KOR signaling decreases excitatory drive to mPFC neurons in a pathway-specific manner via selective KOR-expression, but broadly disinhibits mPFC circuits irrespective of whether the afferent input expresses KOR. This raises the possibility that Dyn / KOR signaling produces opposing effects on integration of excitatory and inhibitory synaptic inputs onto mPFC principal neurons. To determine whether Dyn / KOR signaling regulated KOR-negative and/or KOR-positive afferents to mPFC ensembles, we evoked optogenetic responses from KOR-negative VH and KOR-positive BLA inputs and recorded GCaMP activity in mPFC slices using two-photon imaging (Fig. 6A). Dyn produced a non-significant decrease in BLA-evoked GCaMP activity relative to vehicle-treated slices (Fig. 6B). In contrast, Dyn application enhanced VH-driven Ca^2+^ activity in mPFC networks in comparison to slices treated with vehicle (Fig. 6C). These results suggest that the dual effects of Dyn / KOR signaling on BLA inputs (direct inhibition of BLA terminals and disinhibition of mPFC circuits) versus the singular effect on VH inputs (disinhibition of mPFC circuits) differentially impacts how these two inputs integrate within mPFC neurons.

Subsequently, we probed how Dyn influences integration of excitatory input from KOR-positive and negative sources with local circuit inhibition. We next determined the effects of Dyn bath application on summation of BLA and VH optogenetically evoked postsynaptic potentials (oPSPs). Dyn bath application increased integration of VH-evoked oPSPs (represented as the area under the curve) via inhibition of hyperpolarizing responses associated with train stimulation (Fig. 6D–F). Conversely, Dyn inhibited summation of BLA-evoked oPSPs, by decreasing direct excitation onto pyramidal neurons (Fig. 6D–F). Dyn did not modify VH-evoked EPSCs in voltage clamp where sub-threshold integration with polysynaptic inhibition is not possible (Fig. 1). In current clamp, excitatory inputs can be dampened by polysynaptic inhibition, raising the possibility that Dyn amplification of VH-evoked oPSPs may be due to suppression of GABA_A_ receptor activation. Indeed, Dyn amplification of oPSPs was occluded in cells recorded in the presence of picrotoxin (Fig. 6G), a GABA_A_ receptor antagonist. This demonstrates that Dyn-mediated amplification of VH-evoked oPSPs occurs via disinhibition. Dyn / KOR signaling may shape how synaptic inputs are transformed into action potentials in mPFC neurons. Thus, we determined the effects of Dyn bath application on evoked spiking driven by optogenetic stimulation of BLA and VH inputs. Evoked spiking was driven by excitatory synapses but gated by polysynaptic inhibition. The application of glutamate and GABA_A_ receptor blockers DNQX/AP5 and picrotoxin abolished and enhanced evoked spiking, respectively (Fig. S6A). Dyn significantly enhanced VH-driven spiking in all cells (Fig. 6H, I), an effect that was blocked by nor-BNI (Fig. S6b). Conversely, BLA-driven spiking of mPFC neurons was decreased or showed no change after Dyn application (Fig. 6H, I). Moreover, amplification of VH-evoked firing by Dyn was occluded in slices pretreated with picrotoxin (Fig. 6J). These results indicate that Dyn / KOR signaling differentially shapes input-output transformations into mPFC neurons driven by KOR-negative and positive afferents.

Next, we determined whether Dyn differentially impacted VH and BLA inputs onto the same cell. To this end, we conducted experiments in mice injected with AAV-expressing Chrimson-tdTomato and ChR2-eYFP into the BLA and VH, respectively (Fig. 6K). We used two independent approaches to validate that Chrimson and ChR2-evoked oEPSCs were not mediated via non-selective activation by light sources used to stimulate ChR2 and Chrimson, respectively (Fig. S6C, D). We observed oEPSCs evoked by Chrimson and ChR2 stimulation in individual cells, consistent with the literature demonstrating that VH and BLA inputs converge onto common layer V principal cells [32–36]. Within the same cell, we observed that Dyn produced diametrically opposing effects on VH- and BLA-evoked spiking (Fig. 6L), consistent with the hypothesis that Dyn / KOR signaling toggles how individual mPFC neurons are controlled by converging afferent inputs. In conclusion, Dyn / KOR signaling shapes input / output transformations by limiting mPFC ensemble recruitment by KOR-positive afferents and enhancing incorporation of mPFC ensembles driven by excitatory KOR-negative afferents, including the BLA and VH, respectively (Fig. 6M).

## Discussion

Here we demonstrated that the Dyn / KOR system selectively inhibits (filters) excitatory inputs to the mPFC in a pathway-specific manner via presynaptic KORs. Endogenously released Dyn can act via volume transmission to impact inputs onto both Dyn-positive and Dyn-negative mPFC neurons. Further, we showed that mPFC Dyn / KOR signaling robustly disinhibits mPFC circuits via distinct mechanisms: (1) Potential suppression of feedback inhibition controlled by excitatory mPFC KOR cells, (2) Inhibition of GABA release from subsets of KOR-positive SST- and PV -interneurons and (3) Selective inhibition of excitatory inputs to SST, but not PV- interneurons. Finally, we demonstrate that Dyn / KOR signaling toggles control of mPFC circuits by converging afferents in a pathway-specific manner by presynaptically decreasing excitatory BLA afferents while amplifying the ability of KOR-negative VH inputs to evoke mPFC spiking. Collectively, our study provides a circuit-based model wherein enhanced Dyn / KOR signaling reconfigures the state of mPFC networks and fundamentally changes how KOR-expressing and lacking afferent inputs control pyramidal neurons.

Dyn / KOR signaling inhibits inputs to the mPFC in a pathway-specific manner, including inputs from the BLA, clPFC, and PVT (Fig. 1K). Our results demonstrate that VH afferents to the mPFC are KOR-insensitive, likely due to the absence of KOR mRNA expression in VH output regions. This aligns with previous work demonstrating that systemic KOR activation inhibits BLA to mPFC inputs, but not VH inputs, in anesthetized rats [15]. Our electrophysiological and anatomical studies also suggest that the majority of excitatory inputs onto mPFC neurons are Dyn-insensitive. This provides a mechanism by which mPFC Dyn neurons can selectively influence subsets of excitatory inputs characterized by presynaptic KOR-expression. Indeed, mapping of KOR-positive inputs to the mPFC with KOR-Cre mice failed to label neurons in many projections to the mPFC [22, 23], including the VH. Likewise, excitatory collaterals expressing KOR only represents a fraction of excitatory synapses comprising local circuitry. In response to sustained activity, mPFC Dyn neurons may employ Dyn peptidergic transmission to shape the control of mPFC circuits by KOR afferents. Our findings reveal that endogenous Dyn release functions as a paracrine and/or retrograde signal, limiting incoming excitation from the BLA and likely other KOR-expressing afferents. A subset of mPFC cells expresses Dyn, providing a substrate for the selective influence of KOR-positive afferents by this specific population of mPFC neurons. The impact of endogenous Dyn release extends not only to inputs onto Dyn-positive neurons but also neighboring Dyn-negative neurons, indicating that Dyn may broadly affect mPFC networks through volume transmission. Furthermore, since Dyn and KOR are expressed in distinct neurons, Dyn volume transmission facilitates selective interactions between different cell types in different layers. Sustained activity is necessary for neuropeptide release [11, 37, 38], and similarly, inputs to the mPFC can efficiently transmit information when activity is sustained or repetitive [39, 40]. Therefore, the modulation of different modes of afferent activity by Dyn holds significant implications for how the mPFC processes incoming inputs. Dyn may reduce the gain on KOR-sensitive synapses (e.g., BLA inputs) since the inhibition of glutamate release is not overcome by high-frequency activity (Fig. 6 [15]); Similar to KORs, dopamine D1 receptors sustain inhibition of neurotransmitter release throughout ongoing mPFC afferent activity [41], suggesting that subclasses of GPCRs act to decrease the gain on mPFC networks. Thus, Dyn signaling filters at KOR-positive excitatory afferents regardless of the activity state.

Dyn / KOR signaling may selectively suppress specific inhibitory motifs in mPFC circuits, influencing how pyramidal neurons integrate incoming excitatory inputs in a compartment-specific manner. Our findings demonstrate that Dyn / KOR-mediated disinhibition preferentially inhibits polysynaptic inhibition mediated by SST-interneurons relative to PV-interneurons. Feedforward inhibition conducted by SST neurons is impacted by Dyn / KOR signaling in two ways; (1) via presynaptic KORs that inhibit excitatory inputs onto SST interneurons and (2) via direct inhibition of GABA release from KOR-positive SST interneurons onto principal neurons. In contrast, PV interneuron-mediated feedforward inhibition is only subject to direct inhibition of GABA release from subsets of KOR-positive PV interneurons, since KOR activation does not modify excitatory inputs onto PV neurons. These results establish a framework in which Dyn release differentially affects the balance between SST and PV interneuron-mediated inhibition within the mPFC. SST interneurons play a crucial role in inhibiting the integration of excitatory inputs in the apical dendrites of pyramidal neurons [42]. Importantly, SST-interneurons are preferentially activated by sustained activity, and their inhibitory connections to pyramidal cells exhibit facilitation during ongoing activity [36]. Given the complexity of apical dendritic branching, focal SST dendritic inhibition provides a mechanism for selectively filtering spatial-temporal summation of excitatory inputs within specific branches of the apical dendrite. By suppressing the recruitment and outputs of SST-interneurons, Dyn inhibits SST interneuron-mediated dendritic inhibition, which would otherwise attenuate other incoming afferent inputs. Indeed, we observed that Dyn enhanced the integration of excitatory VH inputs onto pyramidal neurons. In contrast, PV interneurons primarily target the soma of pyramidal cells [36], where they can strongly modulate the final output of the cell regardless of the driving input. Unlike SST neurons, PV cells are rapidly activated by excitatory inputs, and their inhibitory connections onto pyramidal neurons rapidly depress [36]. Since Dyn has limited regulation of PV neuron output compared to SST interneurons, these results suggest that local Dyn release can redistribute the compartmentalization of inhibition. By differentially suppressing SST- and PV-interneuron inhibition, Dyn may also shape the activation of parallel subnetworks of inhibition in response to transient or sustained activity of excitatory afferents. Additionally, Dyn / KOR signaling may contribute to disinhibition by suppressing excitatory KOR-expressing cells that recruit feedback inhibition. Recurrent activity within mPFC microcircuits also promotes the recruitment of inhibitory cortical motifs [5, 7, 8], and Dyn acting on excitatory collaterals from mPFC KOR-positive cells onto neighboring pyramidal neurons may play a crucial role in limiting this process. KOR-mediated disinhibition of mPFC circuits is consistent with previous in-vivo microdialysis findings demonstrating that KOR activation in the mPFC inhibits the ability of a glutamate reuptake blocker to enhance extracellular GABA levels more potently than rises in extracellular glutamate [14]. Dyn has also been shown to inhibit sIPSCs onto pyramidal neurons in the insular cortex [43], suggesting that the Dyn / KOR system may disinhibit other cortical circuits beyond the mPFC. Collectively, Dyn regulation of inhibitory microcircuits and excitatory transmission may forge how mPFC networks orchestrate behavior.

Dyn acting through KORs may modulate how individual mPFC pyramidal neurons process synaptic integration, which, in conjunction with microcircuit interactions, forms the foundation of the cortex’s emergent properties. Emergent properties of the cortex are dynamic, complex physiological processes that include, but are not limited to, sparse encoding of behavioral or internal states at the population level and oscillations that are essential for coordinating activity of single cells across long-range circuits. The role of the Dyn / KOR system in modulating these emergent properties is still unknown. While the Dyn / KOR system has been shown to regulate oscillations in EEG signals in humans and animal models [44–47], it is unclear how much these alterations reflect modulation of oscillations and entrainment of single cell encoding within mPFC circuits. The KOR agonist, Salvinorin A, triggers robust changes in “functional connectivity” across broad cortical networks as assessed by fMRI [48]. Our work shows that Dyn / KOR modulation may in part regulate higher-order processing in mPFC circuits by modifying dendritic computations. Effective summation of dendritic excitatory synaptic potentials triggers active conductances, including dendritic spikes, in apical compartments and promotes burst firing of neurons, which is more likely to occur with strongly weighted synaptic inputs [49–51]. This is significant because active cortical dendrites in layer V pyramidal neurons are believed to be crucial for higher-order processing capabilities of cortical circuits, including the mPFC, essential for top-down control over behavior [52, 53]. By limiting synaptic weights of KOR-expressing afferents, Dyn may reduce dendritic-somatic coupling efficacy, thus gating burst firing. In contrast, by suppressing SST interneuron-driven dendritic inhibition, the Dyn/KOR system allows the weights of excitatory synapses lacking KORs to be amplified and generate dendritic spikes that facilitate burst firing. Pathway-specific control of excitatory synaptic weights by Dyn/KOR signaling shapes how representations encoded by neurons in mPFC afferents are incorporated or filtered into higher-order processing by pyramidal neurons, modulating how the mPFC executes control of behavior. The integration of synaptic inputs provides a cellular mechanism by which representations of behavioral and internal states can be encoded in sparse neuronal ensembles operating through oscillations that synchronize long-range circuits. mPFC SST- and PV-interneurons, as well local recurrent excitatory circuits, are critical for oscillations and associated entrainment of mPFC burst firing during mPFC-dependent cognition [54–57]. Redistribution of compartmentalized inhibition by PV- and SST-interneurons may also underlie the effects of Dyn / KOR signaling in modulating oscillations and higher-order processing in mPFC networks. Our findings that Dyn also inhibits local excitatory collaterals onto pyramidal neurons, also provides an additional mechanism by which KOR signaling may impact sustained firing of mPFC neurons maintained by recurrent excitatory circuits that has been well-documented during cognitive tasks [58–60]. Cognition and emergent properties of the cortex also require coordinated neuromodulation, including potential interactions between Dyn / KOR and dopaminergic signaling. KORs are also expressed on presynaptic DA terminals in the mPFC where they directly inhibit DA release [14]. DA produces complex effects on mPFC circuits, which include regulation of dendritic integration, inhibitory interneurons, and oscillations [5, 41, 61, 62]. In conjunction with pathway-specific regulation of excitation / inhibition balance described in the present study, Dyn / KOR regulation of DA signaling may shape emergent cortical properties gated by DAergic transmission. Taken together, Dyn / KOR regulation of synapse- and cell-specific excitation / inhibition balance is critical for organizing emergent properties of the mPFC that is key for funneling information discretely to downstream targets essential for behavior.

Dysregulated expression of PFC Dyn / KOR and/or KOR availability has been observed in patients who have experienced childhood trauma, as well as chronic exposure to alcohol, opioids, and/or life-long cannabis [16, 18, 63–66]. This has been implicated in mediating cognitive deficits during alcohol and opioid withdrawal. We have shown that mPFC Dyn cell activation and local Dyn release regulates threat-induced defensive behaviors and dynamically shifts neuronal activity in response to threats to a “fear-related” state (Wang et al. in revision). These results are consistent with the proposed model of Dyn / KOR regulation of emergent cortical properties. KOR agonists produce aversive, anxiogenic, and cognitive disruptive effects in humans and animal models, and this in part occurs through actions in mPFC circuits [14–17]. Reduced inhibition and enhanced excitation in cortical circuits is a converging phenotype in various psychiatric disorders associated with mood alterations, psychosis, cognitive deficits, and disruptions in affect (e.g. schizophrenia [55, 67–71]); This is of relevance given that our work demonstrates that Dyn suppresses inhibition and increases excitation of KOR-negative inputs, similar to the converging phenotype described above. These studies are consistent with the hypothesis that dysregulation of mPFC Dyn / KOR control of excitation/inhibition balance caused by chronic drug use and stress may contribute to aforementioned symptoms associated with psychiatric disorders. Future research aimed at identifying how Dyn / KOR signaling within specific mPFC circuit elements shapes processing of motivation, cognition, and perception is warranted to address this knowledge gap. Additionally, the promising results of KOR antagonists in clinical trials for the treatment of anhedonia across diagnoses highlight the potential therapeutic benefits of the mechanistic insights this work provides into the mPFC Dyn / KOR system [72, 73]. Our work also elucidates novel symptom clusters, such as cognitive dysfunction, that can be therapeutically targeted through the Dyn / KOR system. Specifically, targeting KORs that regulate different facets of higher-order circuit function (e.g. dendritic disinhibition) may be useful for personalized and/or symptom-specific therapeutics.

### Supplementary information

Supplemental Figure 1

Supplemental Figure 2

Supplemental Figure 3

Supplemental Figure 4

Supplemental Figure 6

Supplemental materials and methods

Supplemental statistics table
