# Correction: Dynorphin / kappa-opioid receptor regulation of excitation-inhibition balance toggles afferent control of prefrontal cortical circuits in a pathway-specific manner

**DOI:** 10.1038/s41380-023-02368-6

**Published:** 2023-12-19

**Authors:** Hector E. Yarur, Sanne M. Casello, Valerie S. Tsai, Juan Enriquez-Traba, Rufina Kore, Huikun Wang, Miguel Arenivar, Hugo A. Tejeda

**Affiliations:** 1 Unit on Neuromodulation and Synaptic Integration, National Institute of Mental Health, National Institutes of Health, Bethesda, MD USA; 2 NIH Graduate Partnership Program, Washington, DC USA

**Keywords:** Neuroscience, Physiology

Correction to: *Molecular Psychiatry* 10.1038/s41380-023-02226-5, published online 29 August 2023

The article “Dynorphin / kappa-opioid receptor regulation of excitation-inhibition balance toggles afferent control of prefrontal cortical circuits in a pathway-specific manner”, written by Hector E. Yarur, Sanne M. Casello, Valerie S. Tsai, Juan Enriquez-Traba, Rufina Kore, Huikun Wang, Miguel Arenivar & Hugo A. Tejeda, was originally published electronically on the publisher’s internet portal on 29 August 2023 without open access. With the author(s)’ decision to opt for Open Choice the copyright of the article changed on 6 December 2023 to © This is a U.S. Government work and not under copyright protection in the US; foreign copyright protection may apply 2023 and the article is forthwith distributed under a Creative Commons Attribution 4.0 International License, which permits use, sharing, adaptation, distribution and reproduction in any medium or format, as long as you give appropriate credit to the original author(s) and the source, provide a link to the Creative Commons licence, and indicate if changes were made. The images or other third party material in this article are included in the article’s Creative Commons licence, unless indicated otherwise in a credit line to the material. If material is not included in the article’s Creative Commons licence and your intended use is not permitted by statutory regulation or exceeds the permitted use, you will need to obtain permission directly from the copyright holder. To view a copy of this licence, visit http://creativecommons.org/licenses/by/4.0.

