# Supplementary figures and images for: Dynorphin / kappa-opioid receptor regulation of excitation-inhibition balance toggles afferent control of prefrontal cortical circuits in a pathway-specific manner
Source: Mol Psychiatry. 2023 Aug 29;28(11):4801–13. doi: 10.1038/s41380-023-02226-5 (PMC10914606; doi:10.1038/s41380-023-02226-5)

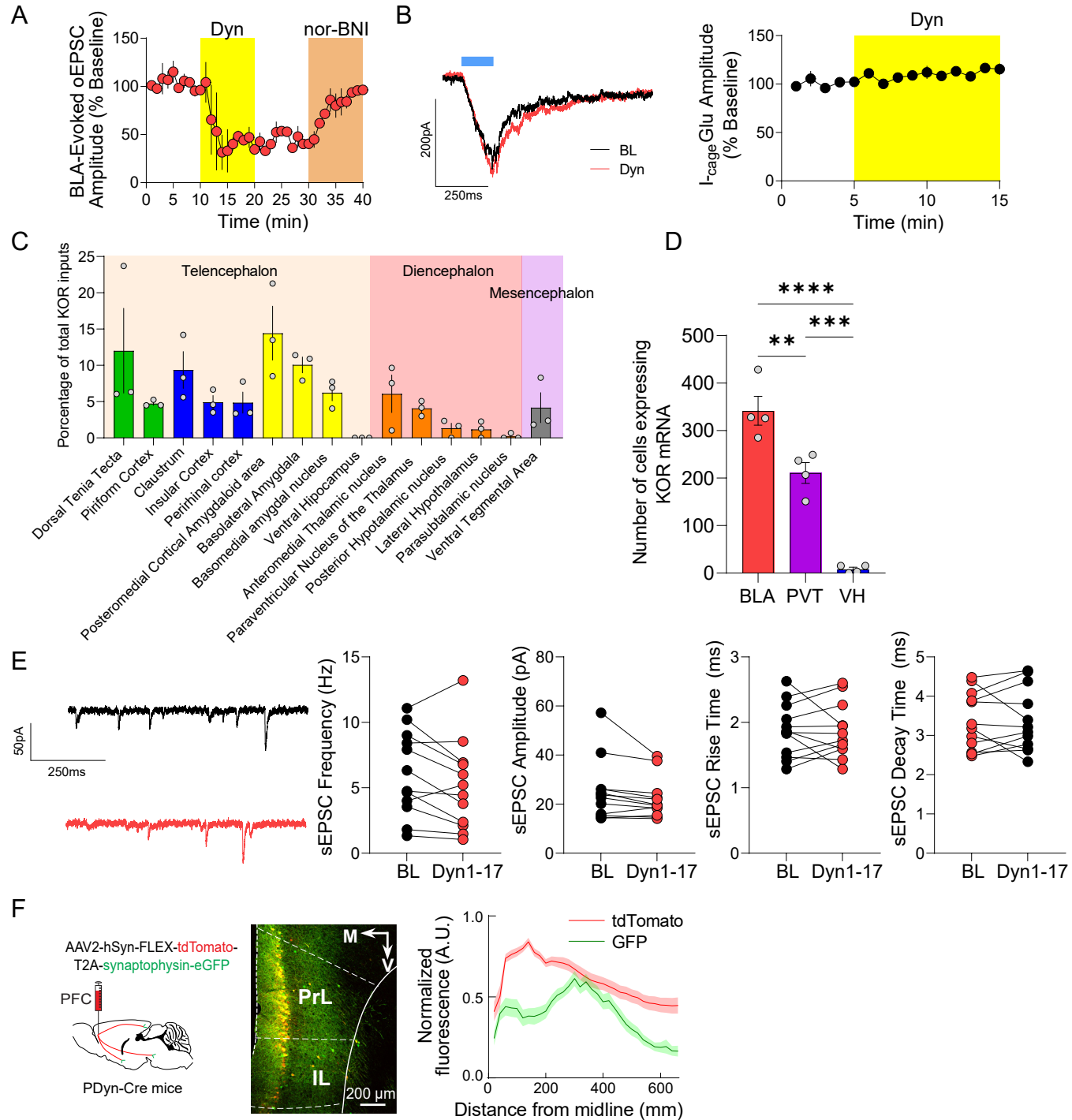

Supplement: Supplementary file 1 — Supplemental Figure 1 [file 41380_2023_2226_MOESM1_ESM.pdf]

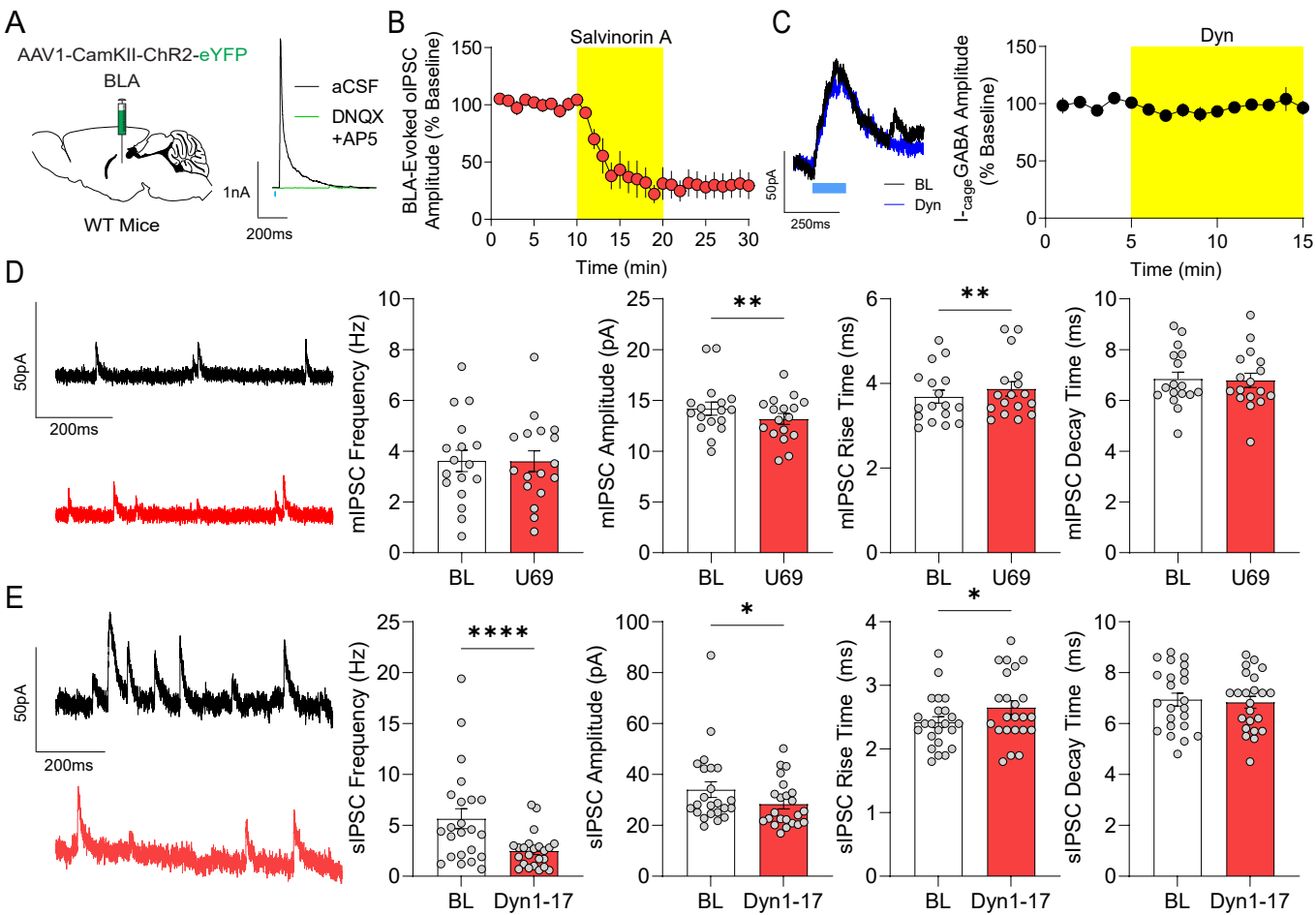

Supplement: Supplementary file 2 — Supplemental Figure 2 [file 41380_2023_2226_MOESM2_ESM.pdf]

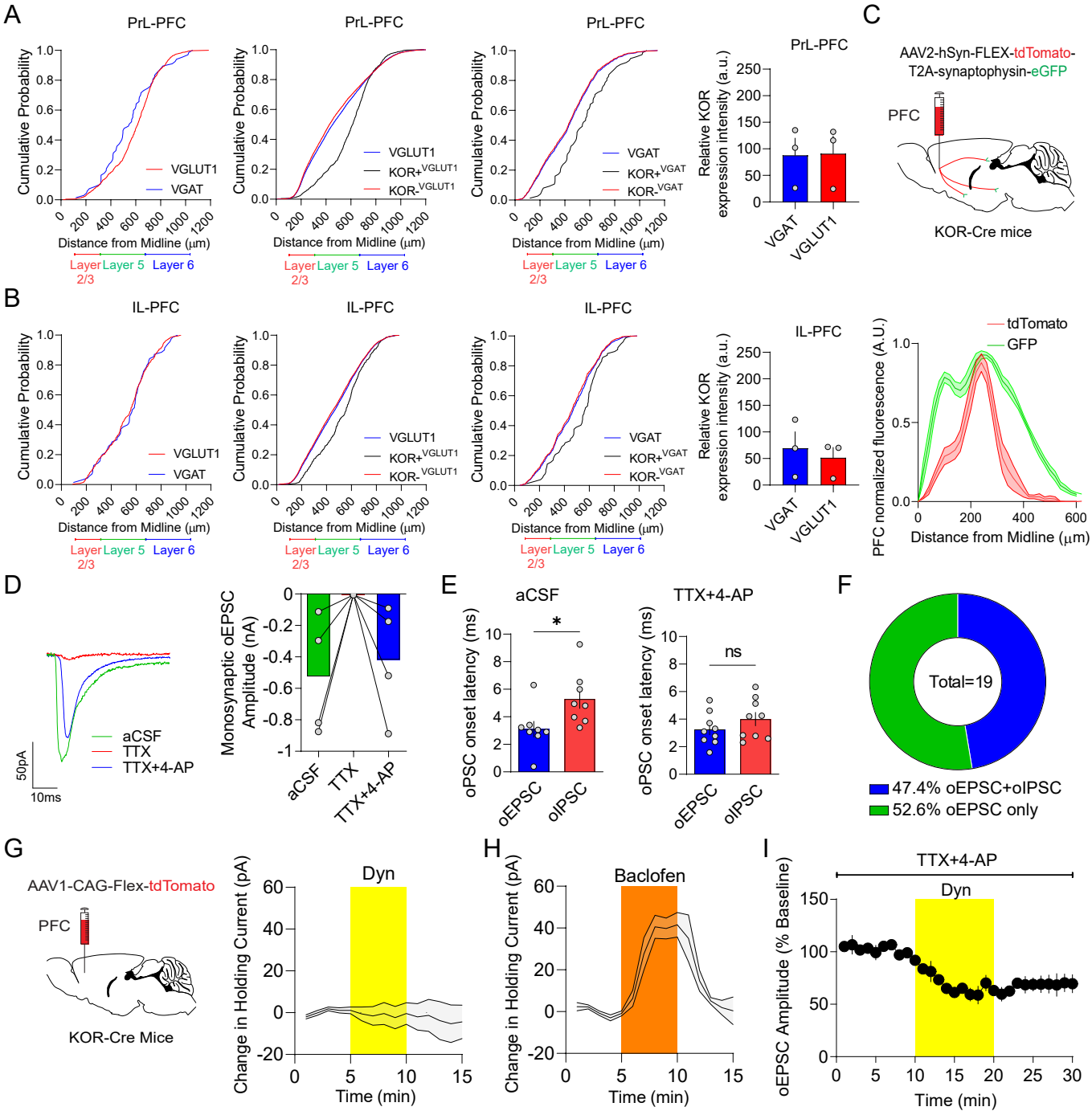

Supplement: Supplementary file 3 — Supplemental Figure 3 [file 41380_2023_2226_MOESM3_ESM.pdf]

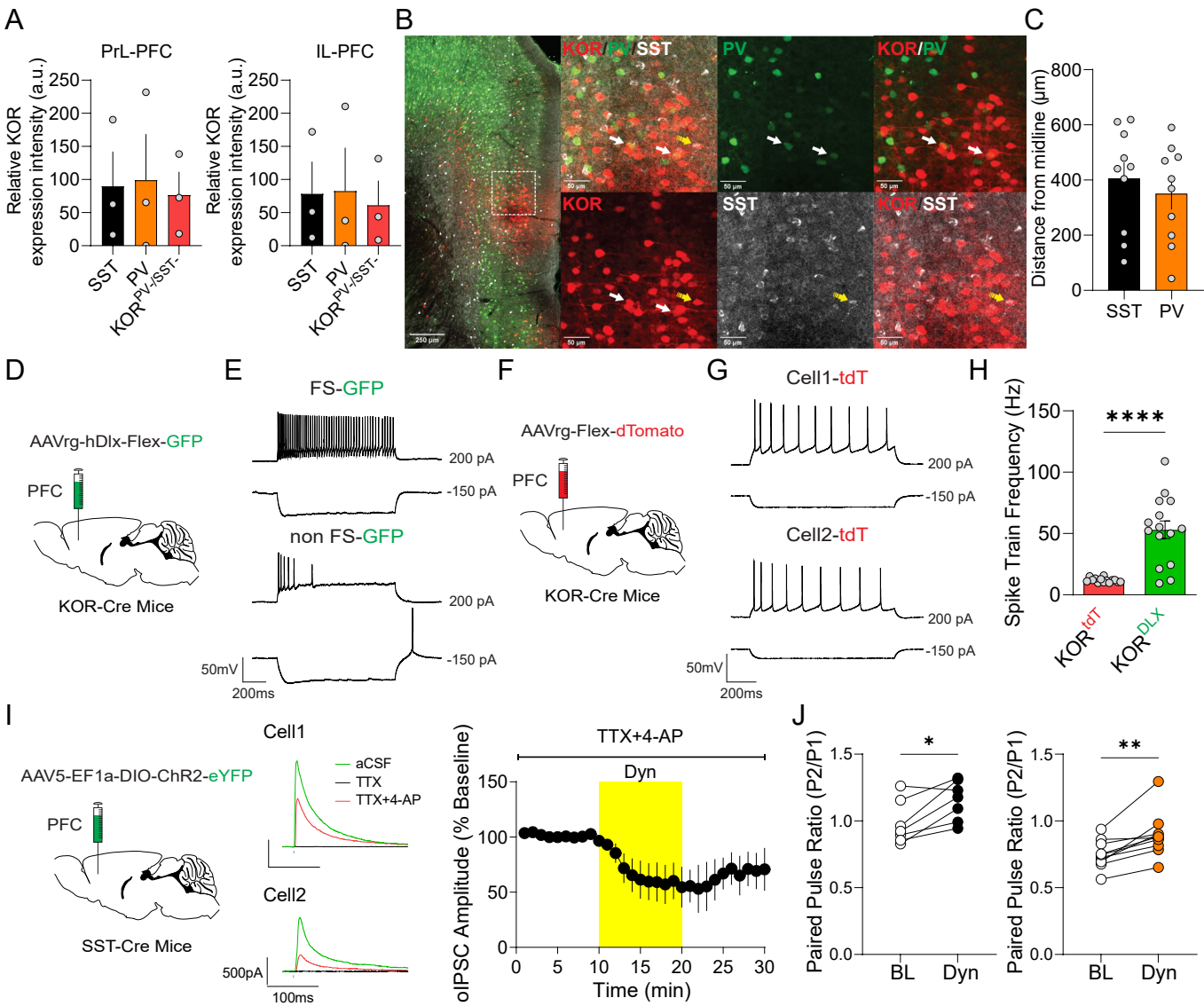

Supplement: Supplementary file 4 — Supplemental Figure 4 [file 41380_2023_2226_MOESM4_ESM.pdf]

**A**

Cell1

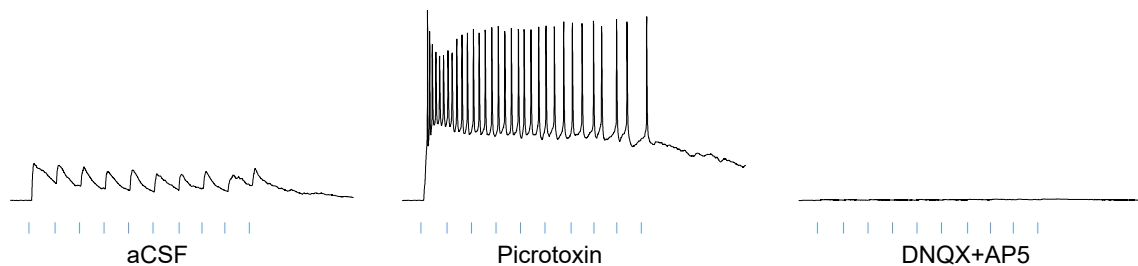

Cell2

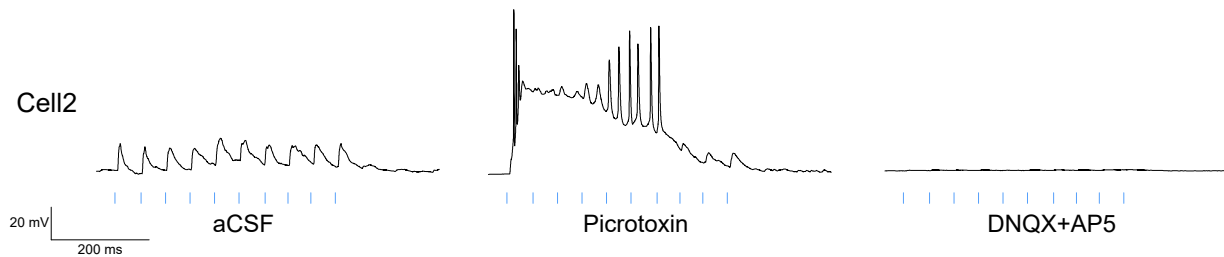**B**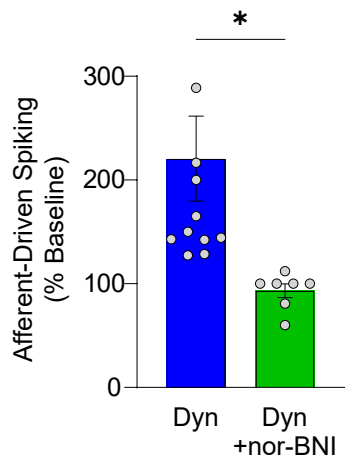**C**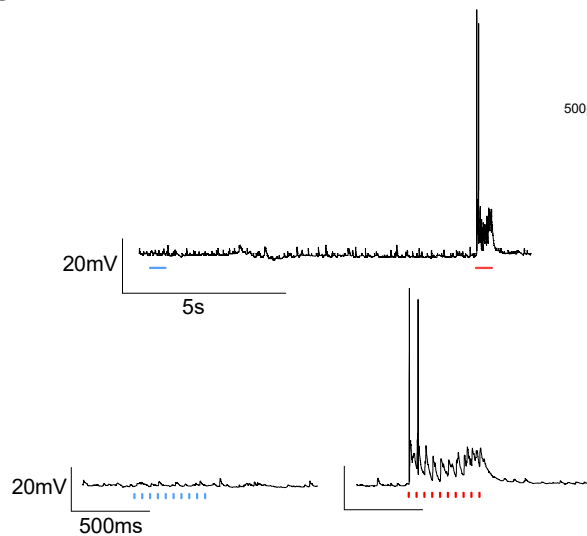**D**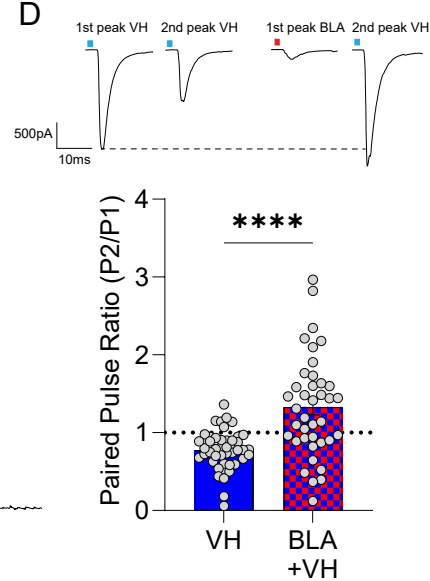

Supplement: Supplementary file 5 — Supplemental Figure 6 [file 41380_2023_2226_MOESM5_ESM.pdf]
