# Supplementary material for: Dynorphin / kappa-opioid receptor regulation of excitation-inhibition balance toggles afferent control of prefrontal cortical circuits in a pathway-specific manner
Source: Mol Psychiatry. 2023 Aug 29;28(11):4801–13. doi: 10.1038/s41380-023-02226-5 (PMC10914606; doi:10.1038/s41380-023-02226-5)
Supplement: Supplementary file 6 — Supplemental materials and methods [file 41380_2023_2226_MOESM6_ESM.docx]

**SUPPLEMENTAL MATERIALS AND METHODS SECTION**

**Animals**

Adult (>postnatal day 60) male and female C57/Bl6J WT, prodynorphin-Cre (PDyn-iCre), somatostatin-Cre (SOM-Cre), parvalbumin-Cre (PV-Cre), kappa opioid receptor-Cre (KOR-Cre), and kappa opioid receptor-loxP (KOR-loxP) mice were used. Mice were housed in humidity and temperature-controlled vivariums using a reverse light cycle with lights off at 7:00 hours and lights on at 19:00 hours. Animals had ad-libitum access to standard laboratory chow and water. Mice were obtained from Jackson Laboratories. All procedures were approved by the National Institute of Mental Health Animal Care and Use Committee.

**Viral Injections**

Mice were anesthetized by ketamine/xylazine cocktail and head-fixed in a stereotaxic frame. For viral injection experiments, subjects were injected with 300-500 nL of virus bilaterally or unilaterally depending on the experiment. The coordinates utilized for the medial prefrontal cortex (mPFC, from bregma: A/P +1.70, M/L +0.3, D/V 2.40 mm), ventral hippocampus (VH, from bregma: A/P 3.27, M/L 3.15, D/V 4.95), paraventricular thalamus (PVT, from bregma: A/P +0.06, M/L -1.58, D/V 3.2 with a 6° degree angle) and basolateral amygdala (BLA, from bregma: A/P -1.65, M/L 3.25, D/V 5.00). Experiments were then performed approximately 4-8 weeks after injection (1 week for retrobeads experiments) to allow sufficient time for expression and trafficking. Acute slices were prepared from most subjects, except for RNAscope or immunohistochemistry experiments where brains were freshly extracted or subjects were perfused, respectively.

**Immunohistochemistry**

Mice were transcardially perfused with phosphate-buffered saline (PBS) followed by 4% paraformaldehyde. Brains were post-fixed in 4% paraformaldehyde for 4 hr and subsequently transferred to PBS. Brain slices (50 µm) containing the mPFC were obtained using a vibratome (Leica). Slices were washed 3 times in PBS, blocked in PBS containing 0.2% Triton-X and 4% bovine serum albumin for 2 hrs. Slices were then incubated with rabbit anti-SOM (1:200) and anti-PV (1:1000) primary antibodies in a blocking solution overnight. The following day sections were washed for 5 min in PBS 3 times. Brain slices were then incubated with donkey anti-mouse (488 nm; 1:500 dilution) and donkey anti-rat secondary (647 nm; 1:200 dilution) for 2 hrs. Slices were then washed for 5 min, 4 times, and mounted prior to imaging using a confocal microscope (Zeiss 780 LSM). Confocal images were taken in z-stacks and acquired using a 20x objective and four channels (DAPI, Alexa-488, Alexa-594, Alexa-647). Laser intensities were kept the same for all images (Green: 2, Red: 15, Far-Red: 23). Maximum intensity z-projection images were subsequently extracted using the image processing software, ImageJ. For quantification of synaptophysin to the mPFC, the ROI image was transformed into an image text file to quantify fluorescence in 20 µm bins, and data were normalized to the maximum value of fluorescence.

**Retrobead retrograde tracing**

C57/Bl6J WT mice were anesthetized and secured on a stereotaxic frame, as described above. Green XI retrobeads (Lumafluor) were unilaterally injected into the mPFC. Retrobeads (150-300 nl) were injected into the mPFC (AP: +1.7 mm; ML: ±0.3 mm; DV: -2.4 mm) and the injector was left in place for 8 min after injection. Brains were removed for RNAscope in situ hybridization 6-7 days after surgery.

**RNAscope in situ hybridization (ISH)**

Brains were rapidly dissected, and flash frozen with isopentane cooled with dry ice for 20 sec and stored at -80°C until sectioning for ISH. Brain slices (16 µm) containing the mPFC, PVT, BLA, and VH were obtained utilizing a Leica CM 3050S cryostat at -20°C and were mounted directly onto microscope slides cleaned with RNAzap, to prevent mRNA degradation. Slides containing ISH sections were stored at -80°C until ISH processing. RNAscope ISH was conducted according to the Advanced Cell Diagnostics user manual and as previously reported [1]. Briefly, slides were fixed in 10% neutral buffered formalin for 20 min at 4°C. Slides were subsequently washed twice for 1 min with PBS, before dehydration with 50% ethanol (5 min), 70% ethanol (5 min), and 100% ethanol (5 min). Slides were incubated in 100% ethanol at -20°C. The following day, slides were dried at room temperature (RT) for 10 min. A hydrophobic barrier was drawn around the sections using a hydrophobic pen and allowed to dry for 10-15 min at RT. Sections were then incubated with Protease Pretreat-4 solution for 20 min at RT. Slides were washed with ddH2O (2 x 1 min), before being incubated with the appropriate probes for 2 hr at 40°C in the HybEZ oven (Advanced Cell Diagnostics). Probes used were purchased from Advanced Cell Diagnostics and are as follows: Mm-Oprk1-C1 (nucleotide target region 256-1457; Accession number NM_001204371.1), Mm-Pvalb-C2 (nucleotide target region 2-885; Accession number NM_013645.3), Mm-Sst-C3 (nucleotide target region 18-407; Accession number NM_009215.1), Mm-Slc17a7-C2 (nucleotide target region 621-1021; Accession number NM_182993.2), Mm-Slc32a1-C3 (nucleotide target region 894-2037; Accession number NM_009508.2), Mm-Pdyn-C2 (nucleotide target region 33-700; Accession number NM_0188863.3). Slides were washed in wash buffer twice for 2 min, prior to being incubated with Amplification 1 buffer at 40°C in the HybEZ oven for 30 min. Slides were subsequently washed in wash buffer twice for 2 min, then incubated with Amplification 2 buffer at 40°C in the HybEZ oven for 15 min. Slides were washed in wash buffer twice for 2 min, prior to being incubated with Amplification 3 buffer at 40°C in the HybEZ oven for 30 min. Slides were subsequently washed in wash buffer twice for 2 min and incubated with Amplification 4-Alt A buffer at 40°C in the HybEZ oven for 15 min. Slides were washed in wash buffer twice for 2 min. DAPI solution was applied to sections at RT for 20 sec. Finally, slides were coverslipped and stored at 4°C until imaging on a confocal microscope (Zeiss 780 LSM). Confocal images were taken in z-stacks and acquired using a 20x objective and four channels (DAPI, Alexa-488, Alexa-550, Alexa-647). Laser intensities were kept the same for all images (Green: 5, Red: 10, Far-Red: 15). Maximum intensity z-projection images were subsequently extracted using the image processing software, ImageJ. A custom pipeline developed in Cell Profiler was used for RNAscope ISH analysis [2].

**Acute brain slice preparation**

Mice were anesthetized with euthanasia (NIH Veterinarian Services) and subsequently decapitated. Brains were rapidly removed and placed in ice-cold NMDG-based cutting solution containing (in mM): 92 NMDG, 20 HEPES, 25 glucose, 30 NaHCO_3_, 2.5 KCl, 1.2 NaPO_4_ saturated, 10 Mg-sulfate, and 0.5 CaCl_2_ with 95% O_2_/5% CO_2_ with an osmolarity of 303-306 mOsm (Wescorp). Following extraction, the brain was rapidly blocked, dried on filter paper, and glued to a platform containing ice-cold NMDG-based cutting solution in a chamber placed within a Leica VT1200 Vibratome. Coronal slices (250 µm thick) containing the mPFC were cut at a speed of 0.07 mm/s. Following slicing, sections were incubated in a chamber containing an NMDG-based cutting solution for 5-10 min at 34°C. Slices were subsequently transferred to a chamber filled with a modified holding aCSF saturated with 95% O_2_/5% CO_2_ containing (in mM): 92 NaCl, 20 HEPES, 25 glucose, 30 NaHCO_3_, 2.5 KCl, 1.2 NaPO_4_, 1 mM Mg-sulfate and 2 mM CaCl_2_ (303-306 mOsm) at room temperature for at least 1 hr. Slices remained in this solution until being transferred to the recording chamber.

**Ex-vivo whole-cell electrophysiology**

Whole-cell patch-clamp electrophysiology studies were performed as previously described [3, 4]. Cells were visualized using IR-DIC optics on an inverted Olympus BX5iWI microscope. For recordings, the recording chamber was perfused with a pump (World Precision Instruments) at a flow rate of 1.5-2.0 ml per minute with aCSF containing (in mM): 126 NaCl, 2.5 KCl, 1.4 NaH_2_PO_4_, 1.2 MgCl_2_, 2.4 CaCl_2_, 25 NaHCO_3_, and 11 glucose (303-305 mOsm). For biophysically isolated oEPSCa and oIPSCs cells were held at -55mV and +10mV respectively. Monosynaptic oEPSCs and oIPSCs evoked by KOR^+^ cell stimulation were isolated with TTX (1 µM) and 4-AP (50 µM). For whole-cell recordings of intrinsic excitability, we utilized glass microelectrodes (3-5 MΩ) containing (in mM): 135 K-gluconate, 10 HEPES, 4 KCl, 4 Mg-ATP, and 0.3 Na-GTP. For oEPSCs and oIPSCs, excitation/inhibition ratios, Rubi-GABA evoked IPSCs, NMI-glutamate evoked EPSCs and NMI-glutamate evoked IPSCs at -55mV and +10 mV we utilized glass microelectrodes (3-5 MΩ) containing (in mM): 117 cesium methanesulfonate, 20 HEPES, 0.4 EGTA, 2.8 NaCl, 5 TEA-Cl, 4 Mg-ATP, 0.4 Na-GTP (280-285 mOsm). ChR2-negative cells were identified by the absence of ChR2 currents evoked by blue light stimulation (<30mW). ChR2 currents were characterized by the presence of sustained, steady-state currents in response to 100 ms blue light stimulation with an onset at the start of the laser pulse. For paired-pulse ratio quantification, light-evoked currents were recorded in response to two light pulses with a 50 ms interstimulus interval. To determine the effects of KOR activation, a ten-minute baseline was collected, and Dyn1-17 (Dynorphin A 1-17) or Salvinorin A was then bath applied for ten minutes and subsequently washed for ten minutes. The last five minutes of baseline and the last five minutes of the drug bath application were used for quantification. To determine the relative engagement of polysynaptic and monosynaptic excitation and inhibition by mPFC KOR-positive neurons, recordings were made in aCSF or TTX and 4AP, isolating polysynaptic and monosynaptic currents, respectively. oEPSCs (0.1 Hz) were recorded at -55 mV until the response was stable. Voltage was then gradually ramped up to +10 mV to biophysically isolate IPSCs. Neurons were voltage-clamped with a Multiclamp 700B amplifier (Molecular Devices). Data were filtered at 2 kHz and digitized at 20 kHz using a 1440A Digidata Digitizer (Molecular Devices). Series resistance (<20 MΩ) was monitored using a -5 mV voltage step. Cells with >20% change in series resistance were discarded from further analysis. For intrinsic excitability, after membrane rupture in voltage clamp, cells were switched to the current clamp configuration without holding current injection. Miniature EPSCs (mEPSCs} were recorded in the presence of 1 µM TTX and picrotoxin (100 µM). Miniature IPSCs (mIPSCs) were collected in the presence of TTX (1 µM), DNQX (10 µM), and D-AP5 (50 µM). For sEPSCs, sIPSCs, mEPSCs, and mIPSCs a ten-minute baseline was collected and U69,593 or Dyn1-17 was subsequently bath applied. The last five minutes of baseline and the last five minutes of drug were used for quantification and were counted manually utilizing Minianalysis software (Synaptosoft). Change in holding current by Dyn1-17 was evaluated at -60 mV, a positive control was used Baclofen 10µM. MNI-glutamate was bath applied at 50 µM in 10 ml of ACSF. Experiments at -55 mV and +10 mV were performed to isolate uEPSC and uIPSC, respectively. DNQX+AP5 was added to demonstrate that glutamate uncaging-evoked IPSCs were driven by glutamate receptor activation. Glutamate uncaging was achieved using 150 ms pulses of UV light stimulation with variable light intensity in order to obtain a stable baseline. Intrinsic excitability was assessed by applying hyperpolarizing and depolarizing current steps (25 pA steps: 1-sec duration) and measuring the change in voltage and action potential firing. For experiments determining the effects of Dyn1-17 on synaptically-driven mPFC neuron spiking, whole-cell recordings were performed using a potassium gluconate-based internal solution. Optogenetic synaptically-driven spiking was evoked using a 10 pulse 20 Hz stimulation train delivered every 20 sec. For synaptic voltage clamp experiments, light intensity was adjusted to optically evoke PSCs at approximately half the maximally-evoked PSCs amplitude to permit the detection of increases or decreases in evoked PSCs. For current clamp recordings light intensity was adjusted to evoke 3-7 spikes to likewise permit increases and decreases in afferent-driven action potentials. Cells, where afferent stimulation failed to drive action potentials, were used for sub-threshold PSPs experiments. A five-minute baseline was collected, and Dyn1-17 was subsequently bath applied. The last two minutes of baseline and the last two minutes of drug were used for quantification. For all experiments, cells that underwent a 20% increase in access resistance or greater were excluded from analysis.

**Two-Photon Imaging**

Imaging was performed by using an upright FVMPE-RS multiphoton microscope (Olympus) equipped with an InSight DS Dual-OL fs-laser system (Spectra-Physics) and equipped with 40X, 0.8 NA water-immersion objective (Olympus). For 2PLSM, 940 nm light was used to excite the genetically-encoded calcium sensor jGCaMP7f. Reference frame scans were taken between each acquisition to correct for small spatial drift of the preparation over time. Optogenetic synaptically-driven spiking was evoked by a 10 pulse 20 Hz train stimulation delivered every 30 sec by a 635 nm laser directed at the slice containing the ROI being imaged with the microscope. The stimulation intensity used was approximately 60 mW. To measure Ca^2+^ signals, green fluorescence was collected at 15 Hz in a full field scan using a 512x512 resonant scanner. Ca^2+^ signals were quantified as changes in fluorescence relative to the baseline time. Images were analyzed offline with ImageJ (NIH). Data are presented as the area under the curve in fluorescence with respect to the baseline period.

**Drugs**

Drugs were dissolved in aCSF or water. Drugs were purchased from Sigma Aldrich, Tocris, or generously provided by the NIDA Drug Supply Program.

**Statistics**

Statistics were computed using GraphPad Prism. No randomization method was used in this study. Experimenters were not blinded to experimental conditions.

**REFERENCE**

1. Tejeda HA, Counotte DS, Oh E, Ramamoorthy S, Schultz-Kuszak KN, Backman CM *et al.* Prefrontal cortical kappa-opioid receptor modulation of local neurotransmission and conditioned place aversion. *Neuropsychopharmacology* 2013; **38**(9)**:** 1770-1779.

2. Erben L, Buonanno A. Detection and Quantification of Multiple RNA Sequences Using Emerging Ultrasensitive Fluorescent In Situ Hybridization Techniques. *Curr Protoc Neurosci* 2019; **87**(1)**:** e63.

3. Tejeda HA, Wu J, Kornspun AR, Pignatelli M, Kashtelyan V, Krashes MJ *et al.* Pathway- and Cell-Specific Kappa-Opioid Receptor Modulation of Excitation-Inhibition Balance Differentially Gates D1 and D2 Accumbens Neuron Activity. *Neuron* 2017; **93**(1)**:** 147-163.

4. Pignatelli M, Tejeda HA, Barker DJ, Bontempi L, Wu J, Lopez A *et al.* Cooperative synaptic and intrinsic plasticity in a disynaptic limbic circuit drive stress-induced anhedonia and passive coping in mice. *Mol Psychiatry* 2021; **26**(6)**:** 1860-1879.

**SUPPLEMENTAL FIGURE LEGENDS**

**SF1.**

1. Time course of the effect of Dyn on oEPSC amplitude in mPFC pyramidal neurons evoked by ChR2 stimulation of the BLA to mPFC pathway. Dyn-mediated inhibition of oEPSCs in mPFC cells was reversed by bath application of nor-BNI.
2. Representative EPSCs recorded in mPFC pyramidal neurons evoked by MNI-Glutamate uncaging during baseline (black) and after Dyn (red). Dyn did not have an effect on the amplitude (expressed as a percentage of baseline) of the EPSC evoked by MNI-Glutamate in the mPFC.
3. Percentage of KOR-expressing cells that project to PFC.
4. Number of cells expressing KOR mRNA in the PVT, BLA, and VH.
5. Dyn did not modulate sEPSC frequency or amplitude in mPFC cells, baseline (black), or Dyn (red). sEPSC frequency, amplitude, rise time, and decay time at baseline and during Dyn application.
6. Schematic and representative image of AAV-hSyn-FLEX-TdTomato-T2A-Synapsin-eGFP expression in the mPFC of PDyn-Cre mice. Normalized distribution of PFC Dyn cell arborization (tdTomato) and putative PFC Dyn synapses (eGFP lacking tdTomato) across layers of the PFC.

**SF2.**

1. WT mice were injected bilaterally with AAV1-CaMKII-ChR2-eYFP in BLA of WT mice. Representative oIPSCs traces recorded in PFC from BLA during baseline (dark traces) and after DNQX+AP5 (green traces) are shown.
2. Time course of the effect of KOR activation with Salvinorin A (1 µM) on the amplitude of oIPSCs in the mPFC of animals expressing ChR2-eYFP in the BLA.
3. Representative oIPSCs evoked by Rubi-GABA uncaging in mPFC neurons. GABA uncaging-evoked IPSCs during baseline (black) and after Dyn1-17 (blue) are shown. Dyn did not have an effect on the amplitude of oIPSCs evoked by Cage-GABA in PFC.
4. Representative mIPSC traces during baseline (black) or U69,593 application (red). Comparison of mIPSC frequency, amplitude, rise time, and decay time between baseline and U69,593 application.
5. Representative sIPSC traces of baseline (black) or Dyn1-17 (red). Comparison of sIPSC frequency, amplitude, rise time, and decay time between baseline and Dyn.

**SF3.**

1. Cumulative probability of VGAT (blue) and VGLUT1 (red) mRNA across PrL-PFC layers. Cumulative probability of KOR mRNA expression with (black) and without (red) VGLUT1 across PrL-PFC layers. Cumulative probability of KOR mRNA expression with (black) and without (red) VGAT across PrL-PFC layers. Relative KOR mRNA expression in cells containing VGAT and VGLUT1 mRNA in PrL-PFC. Data are mean ± SEM.
2. Cumulative probability of VGAT (blue) and VGLUT1 (red) mRNA across IL-PFC layers. Cumulative probability of KOR mRNA expression with (black) and without (red) VGLUT1 across IL-PFC layers. Cumulative probability of KOR mRNA expression with (black) and without (red) VGAT across IL-PFC layers. Relative KOR mRNA expression in cells containing VGAT and VGLUT1 mRNA in IL-PFC. Data are mean ± SEM.
3. Schematic depicting AAV-hSyn-FLEX-TdTomato-T2A-Synapsin-eGFP expression in the mPFC of KOR-Cre mice. Normalized distribution of mPFC KOR cell arborization (tdTomato) and putative KOR synapses within the mPFC (eGFP signal lacking tdTomato) across mPFC layers.
4. Representative traces of oEPSC from KOR cells in the PFC that make excitatory monosynaptic connections. Mean monosynaptic oEPSC amplitude in the presence of aCSF, TTX and TTX+4-AP. Data are mean ± SEM.
5. Mean aCSF and monosynaptic (TTX+4-AP) oEPSC and oIPSC latency. Data are mean ± SEM.
6. Pie chart showing the proportion of evoked responses consisting of oEPSC+IPSC and oEPSC from KOR-positive neurons.
7. KOR-Cre mice were injected bilaterally with AAV1-CAG-Flex-tdTomato into PFC. Time course of the effect of KOR activation with Dyn on change in holding current on mPFC KOR neurons.
8. Time course of the effect of GABA-B activation with baclofen (1 µM) on change in holding current in mPFC principal neurons.
9. The amplitude of evoked monosynaptic oEPSCs from KOR-positive neurons onto mPFC pyramidal neurons are inhibited by Dyn.

**SF4.**

1. Relative KOR mRNA expression in cells containing or lacking SST and PV mRNA in mPFC.
2. KOR-Cre mice were injected unilaterally with AAVrg-FLEX-tdTomato into the mPFC. Representative images show colocalization of tdTomato-labeled mPFC KOR-expressing cells with PV- (green) and SST-immunoreactivity in a sparse population of tdTomato-positive cells (white).
3. Relative distance from midline of KOR-positive tdTomato cells colocalized with PV or SST-immunoreactivity, which is similar to the distribution KOR mRNA-positive PV and SST-mRNA positive cells as assessed by *in-situ* hybridization in WT mice.
4. KOR-Cre mice were injected bilaterally with AAVrg-hDlx-Flex-GFP into the mPFC to label KOR-positive inhibitory interneurons.
5. Representative traces at different current steps showing intrinsic properties consistent with a fast-spiking interneuron (top) and a non-fast spiking, non-regular spiking interneuron (bottom).
6. Schematic showing bilateral injection of AAVrg-Flex-tdTomato into the mPFC of KOR-Cre mice to label all KOR-positive neurons.
7. Representative traces of intrinsic excitability at different current steps of two tdTomato positive cells, consistent with regular firing pyramidal neurons.
8. Comparison of spike train frequency between KOR-excitatory and inhibitory cells (KOR-tdT and KOR-Dlx).
9. Schematic depicting SST-Cre mice injected bilaterally with AAV5-EF1α-DIO-ChR2-eYFP into the mPFC. Representative oIPSCs traces recorded from two cells in PFC during baseline (green traces), TTX (dark traces) and TTX/4-AP (red traces) are shown. Time course of monosynaptic oIPSCs in the presence of TTX/4-AP demonstrating the inhibitory effect Dyn (1 µM) application from animals expressing ChR2-eYFP in SST-positive mPFC neurons.
10. Comparison of the effect of KOR activation by Dyn on the paired-pulse ratio of oIPSC evoked from SST-positive (black) and PV-positive (orange) neurons in the mPFC.

**SF6.**

1. Representative traces of two neurons demonstrating that synaptically driven action potential firing by train stimulation of VH inputs (10 pulses at 20 Hz) is under the control of GABAergic inhibition. Evoked spiking during baseline, in the presence of picrotoxin, and after DNQX+APV application. Blue lines indicate timing of LED pulses.
2. nor-BNI antagonism of Dyn-mediated disinhibition of afferent driven spiking.
3. Traces showing that BLA afferents expressing Chrimson in mice that were not expressing ChR2 in the VH were activated by red-, but not blue-shifted light.
4. (Top) Representative traces of paired-pulse stimulation of VH inputs (left) or BLA and VH inputs (right). (Bottom) Graph showing paired-pulse experiments where selective activation of VH inputs with blue light was associated with paired-pulse depression, but no depression of VH-evoked oEPSC was observed when red-shifted light activation of BLA inputs was used as a prepulse.

**KEY RESOURCES TABLE**

| REAGENT | SOURCE | IDENTIFIER |
| --- | --- | --- |
| ANTIBODIES | | |
| Anti-PV antibody mouse monoclonal (clone Parv-19) | MilliporeSigma | Cat# P3088 |
| Anti-Somatostatin Antibody, clone YC7 | MilliporeSigma | Cat# MAB354 |
| Alexa Fluor® 488 AffiniPure Donkey Anti-Mouse IgG (H+L) | Life Technologies | Cat# A21202 |
| Alexa Fluor® 647 AffiniPure Donkey Anti-Rat IgG (H+L) | Jackson ImmunoResearch | Cat# 712-605-153 |
|  |  |  |
| BACTERIAL AND VIRUS STRAINS | | |
| AAV2/9-phSyn1(S)-Flex-tdTomato-T2A-SypEGFP-WPRE | Boston Children’s Hospital Viral Core | NA |
| AAV1-Syn-ChrimsonR-tdT | UNC | Lot# AV6549 |
| AAV1-CamKII-ChR2-eYFP | Addgene/UNC | Cat# 26969 |
| AAV5-EF1α-DIO-ChR2-eYFP | UNC vector core | Lot# AV4313-2A |
| AAV1-EF1α-DO-ChR2-eYFP | NIDA GEVVC | Lot# AAV897 |
| AAV5-Syn-Flex-HM3Dq-mCherry | Addgene | Cat# 44361 |
| AAVrg-FLEX-tdTomato | Addgene | Cat# 28306 |
| AAV5-hSyn-Cre-P2A-tdTomato | Addgene | Cat# 107738 |
| AAVrg-hDlx-Flex-GFP | Addgene | Cat# 83895 |
| AAVrg-mDLX-GFP | Addgene | Cat# 83895 |
| AAV9-syn-jGCaMP7f-WPRE | Addgene | Cat# 104488 |
| AAV1-CAG-Flex-tdTomato | UNC | Lot# AV5328B |
|  |  |  |
| CHEMICALS, PEPTIDES, AND RECOMBINANT PROTEINS | | |
| TTX | Tocris | Cat# 1069 |
| 4AP | Tocris | Cat# 0940 |
| DNQX Disodium Salt | Abcam | Cat# ab120169 |
| D-AP5 | Abcam | Cat# ab120003 |
| Picrotoxin | Abcam | Cat# ab120315 |
| Dynorphin A 1-17 | NIDA | Cat# MPSP-015 |
| 5-met-Enkephalin | NIDA | Cat# MPSP-029 |
| Nor-BNI | Tocris | Cat# 0347 |
| NMI-Glutamate | Tocris | Cat# 1490 |
| Rubi-GABA | Tocris | Cat# 3400 |
| Salvinorin A | NIDA | Cat# NOCD-099 |
| R-Baclofen | Tocris | Cat# 0796 |
|  |  |  |
| EXPERIMENTAL MODELS: ORGANISMS/STRAINS | | |
| Mouse: C57BL/6J | The Jackson Laboratory | Strain #: 000664 |
| Mouse: PDyn-Cre | The Jackson Laboratory | Strain #: 027958 |
| Mouse: KOR-Cre | The Jackson Laboratory | Strain #: 035045 |
| Mouse: KOR-Loxp | William Carlezon (Harvard University) |  |
| Mouse: SOM-Cre | The Jackson Laboratory | Strain #: 028864 |
| Mouse: PV-Cre | The Jackson Laboratory | Strain #: 008069 |
|  |  |  |
| SOFTWARE AND ALGORITHMS | | |
| Fiji (ImageJ) | Schneider et al., 2012 | https://imagej.net/software/fiji/ |
| GraphPad Prism 9 | GraphPad software | https://www.graphpad.com/ https://www.moleculardevices.com/  products/axon-patch-clamp-system scientific-software/prism/ |
| Clampex and Clampfit 11 | Molecular Devices | https://support.moleculardevices.com/s/article/Axon-pCLAMP-11-Electrophysiology-Data-Acquisition-Analysis-Software-Download-Page |
| RStudio |  | https://www.rstudio.com/ |
| Adobe Illustrator | Adobe | https://www.adobe.com/products/illustrator.html |
| Minianalysis | Synaptosoft Inc | http://www.synaptosoft.com/MiniAnalysis/ |

**Table 1. Key materials table containing information about the essential resources utilized in the project.**
