# Supplementary material for: Dynorphin / kappa-opioid receptor regulation of excitation-inhibition balance toggles afferent control of prefrontal cortical circuits in a pathway-specific manner
Source: Mol Psychiatry. 2023 Aug 29;28(11):4801–13. doi: 10.1038/s41380-023-02226-5 (PMC10914606; doi:10.1038/s41380-023-02226-5)
Supplement: Supplementary file 7 — Supplemental statistics table [file 41380_2023_2226_MOESM7_ESM.docx]

| Figure | | Animal and cell count | Statistics | Descriptive Statistic |
| --- | --- | --- | --- | --- |
| 1 | B | PVT, n = 9 cells in 3 male and 2 female mice  BLA, n = 12 cells in 4 male and 2 female mice  clPFC, n = 8 cells in 2 male and 2 female mice  VH, n = 6 cells in 2 male and 3 female mice |  |  |
|  | C |  | One-way ANOVA.  F_(3, 30)_ = 11.95, *p* < 0.0001; **p* = 0.0471, ****p* = 0.0009, *****p* < 0.0001 | PVT = 50.30 ± 6.537  BLA = 36.60 ± 4.761  clPFC = 25.59 ± 5.591  VH = 2.255 ± 1.887 |
|  | E | n = 2 male and 1 female mice | One-way ANOVA.  F_(2, 6)_ = 49.32, *p* = 0.0002; **p* = 0.0168,  ***p* = 0.0026, ****p* = 0.0002 | PVT = 4.077 ± 0.602  BLA = 10.07 ± 1.095  VH = 0.000 ± 0.000 |
|  | H | n = 2 male and 2 female mice |  | PVT = 0.4225 ± 0.0709  BLA = 0.4900 ± 0.0656  VH = 0.0137 ± 0.0084 |
|  | J | PDyn-positive, n = 7 cells in 4 male and 1 female  PDyn-negative, n = 8 cells in 4 male and 2 female mice  nor-BNI, n = 5 cells in 3 male and 1 female mice | One-way ANOVA.  F_(2, 17)_ = 6.22, *p* = 0.0051; ***p* = 0.0074;  * *p* = 0.042 | PDyn-positive = 45.70 ± 10.37  PDyn-negative = 32.96 ± 6.084  nor-BNI = 4.505 ± 3.840 |
| S1 | A | n = 3 cells in 2 male mice | One-way ANOVA.  F_(2, 109)_ = 0.508, *p* = 0.603 |  |
|  | B | n = 6 cells in 1 male and 4 female mice |  |  |
|  | C | n = 2 male and 1 female mice | One-way ANOVA.  F_(14, 30)_ = 3.83, *p* = 0.001 |  |
|  | D |  | One-way ANOVA.  F_(2, 9)_ = 59.52, *p* < 0.0001, ***p* = 0.0055, ****p* = 0.0003, *****p* < 0.0001 |  |
|  | E | n = 12 cells in 6 male and 5 female mice | Paired t-test.  Frequency, t_(11)_ = 1.97, *p* = 0.0735  Amplitude, t_(11)_ = 1.98, *p* = 0.0729  Rise time, t_(11)_ = 0.04, *p* = 0.9635  Decay time, t_(11)_ = 0.008, *p* = 0.9936 |  |
|  | F | n = 3 male and 2 female mice |  |  |
| 2 | B | PVT, n = 9 cells in 4 male and 1 female  BLA, n = 9 cells in 3 male and 3 female mice  clPFC, n = 10 cells in 2 male and 2 female mice  VH, n = 8 cells in 1 male and 2 female mice |  |  |
|  | C |  | Two-way ANOVA repeated measures; PSC x pathway interaction.  F_(3, 60)_ = 4.37, *p* = 0.0075; PSC main effect; F_(1, 60)_ = 91.94, *p* < 0.0001 | oIPSC  PVT = 88.88 ± 6.304  BLA = 72.85 ± 5.279  clPFC = 47.22 ± 8.045  VH = 68.99 ± 5.474  oEPSC  PVT = 50.30 ± 6.537  BLA = 37.48 ± 4.368  clPFC = 25.59 ± 5.591  VH = 3.181 ± 1.314 |
|  | E | MNI-glutamate, n = 8 cells in 1 male and 4 female mice  nor-BNI, n = 6 cells in 1 male and 3 female | Two-way ANOVA repeated measures; Time x drug interaction.  F_(14, 164)_ = 11.29, *p* < 0.0001  Percent inhibition  t_(12)_ = 5.33, *p* = 0.0002 | MNI-glutamate = 65.92 ± 9.105  nor-BNI = 3.932 ± 5.522 |
|  | F | WT, n = 5 cells in 2 male and 1 female mice  KOR-loxP, n = 11 cells in 4 male and 1 female mice | Two-way ANOVA repeated measures; Time x virus interaction.  F_(14, 194)_ = 8.44, *p* < 0.0001).  Percent inhibition  t_(14)_ = 5.01, *p* = 0.0002 | WT = 49.00 ± 6.962  KOR-loxP = 8.884 ± 4.386 |
| S2 | B | n = 6 cells in 2 male and 4 female mice |  |  |
|  | C | n = 6 cells in 3 female mice |  |  |
|  | D | n = 17 cells in 4 male and 4 female mice | Paired t-test.  Frequency, t_(16)_= 0.08, *p* = 0.9360  Amplitude, t_(16)_ = 3.22, *p* = 0.0053  Rise time, t_(16)_ = 3.51, *p* = 0.0029  Decay time, t_(16)_= 0.38, *p* = 0.7020 |  |
|  | E | n = 23 cells in 2 male and 9 female mice | Paired t-test.  Frequency, t_(22)_ = 4.92, *p* < 0.0001  Amplitude, t_(22)_ = 2.72, *p* = 0.0123  Rise time, t_(22)_ = 2.24, *p* = 0.0354  Decay time, t_(22)_ = 0.74, *p* = 0.4659 |  |
| 3 | B | n = 2 male and 2 female mice | K-S Test; D=12.09, *p*<0.0001 |  |
|  | D | n = 2 male and 1 female mice | Proportion of KOR cells in VGAT or VGLUT1  t_(4)_= 2.63, *p* = 0.0582  Proportion of total KOR cells  t_(4)_= 30.21, *p* < 0.0001 | Proportion of KOR cells in VGAT or VGLUT1  VGAT = 5.701 ± 1.715  VGLUT1 = 10.80 ± 0.905  Proportion of total KOR cells  VGAT = 6.403 ± 2.345  VGLUT1 = 94.09 ± 1.711 |
|  | G | TTX+4-AP, n = 8 cells in 2 male and 2 female  aCSF, n = 9 cells in 3 male and 2 female mice | t_(15)_= 4.35, *p* = 0.0006 | TTX+4-AP = 1.955 ± 0.4321  aCSF = 0.1802 ± 0.0382 |
|  | I | Cre-ON, n = 4 cells in 1 male and 2 female mice  Cre-OFF, n = 7 cells in 3 male and 1 female | Two-way ANOVA repeated measures; Time x virus interaction.  F_(29, 259)_ = 4.08, *p* < 0.0001  Percentage inhibition  t_(9)_= 3.48, *p* = 0.0069 | Cre-ON = 50.12 ± 11.58  Cre-OFF = 14.57 ± 4.30 |
|  | K | Cre-ON, n = 5 cells in 2 male and 1 female mice  Cre-OFF, n = 5 cells in 5 male mice | Two-way ANOVA repeated measures; Time x virus interaction.  F_(29, 2232)_ = 0.45, *p* = 0.9934).  Percentage inhibition  t_(8)_= 0.36, *p* = 0.7252 | Cre-ON = 57.93 ± 8.280  Cre-OFF = 53.94 ± 7.177 |
| S3 | A | n = 2 male and 1 female mice | t_(4)_= 0.06, *p* = 0.9498 |  |
|  | B | n = 2 male and 1 female mice | t_(4)_= 0.48, *p* = 0.6524 |  |
|  | C | n = 2 male and 2 female mice | Two-way ANOVA repeated measures; Fluorophore x distance interaction.  F_(31, 486)_ = 7.43, *p* < 0.0001 |  |
|  | D | n = 4 cells in 2 male and 2 female mice | t_(25)_=1.46, *p*=0.1563 |  |
|  | E | aCSF, n = 16 cells in 2 male and 2 female mice  TTX+4-AP, n = 18 cells in 2 male and 2 female mice | aCSF, t_(14)_=2.46, *p*=0.0277  TTX+4-AP, t_(16)_=1.19, *p*=0.2495 |  |
|  | G | n = 10 cells in 4 male mice |  |  |
|  | H | n = 10 cells in 4 male mice |  |  |
|  | I | n = 4 cells in 3 male mice |  |  |
| 4 | B | n = 14 cells in 2 male and 2 female mice | One-way ANOVA.  F_(12, 377)_ = 2.097, *p* = 0.016 |  |
|  | D | n = 2 male and 1 female mice | t_(4)_= 1.25, *p* = 0.2778 | SST = 20.35 ± 2.854  PV = 11.60 ± 6.358 |
|  | E | n = 2 male and 1 female mice | t_(4)_= 4.69, *p* = 0.0093 | SST = 9.804 ± 1.464  PV = 2.166 ± 0.705 |
|  | G | aCSF, n = 7 cells in 3 male and 1 female mice  norBNI, n = 3 cells in 1 male and 1 female mice | Two-way ANOVA repeated measures; Time x virus interaction.  F_(29, 232)_ = 5.98, *p* <0.0001 |  |
|  | I | aCSF, n =10 cells in 3 male and 2 female  norBNI, n = 6 cells in 1 male and 2 female mice | Two-way ANOVA repeated measures; Time x virus interaction.  F_(29, 406)_ = 8.54, *p* <0.0001 |  |
|  | J | SST, n = 7 cells in 3 male and 1 female mice  PV, n =10 cells in 3 male and 2 female | t_(15)_= 2.75, *p* = 0.0147 | SST = 57.55 ± 1.760  PV = 43.73 ± 3.974 |
| S4 | A | n = 2 male and 1 female mice | PrL-mPFC, One-way ANOVA.  F_(2, 6)_ = 0.044, *p* = 0.9565  IL-mPFC, One-way ANOVA.  F_(2, 6)_ = 0.049, *p* = 0.9524 |  |
|  | B | n = 2 male and 1 female mice |  |  |
|  | C |  | t_(18)_= 0.67, *p* = 0.5104 |  |
|  | H | KOR-tdT, n = 14 cells in 4 male  KOR-Dlx, n = 19 cells in 1 male and 2 female | t_(27)_= 5.52, *p* < 0.0001 |  |
|  | I | n = 4 cells in 2 male mice |  |  |
|  | J |  | SST-positive, t_(6)_= 3.46, *p* = 0.0134  PV-positive, t_(9)_= 4.37, *p* = 0.0018 |  |
| 5 | B | FSI, n = 6 cells in 2 male and 2 female  non-FSI, n = 5 cells in 2 male and 2 female mice | Two-way ANOVA repeated measures; Time x virus interaction.  F_(29, 261)_ = 3.64, *p* <0.0001 |  |
|  | C |  | t_(9)_= 3.02, *p* = 0.0143 | Non-FS = 56.07 ± 13.20  FS = 13.34 ± 6.85 |
|  | E | SST, n = 10 cells in 5 male  PV, n = 13 cells in 3 male and 2 female mice | Two-way ANOVA repeated measures; Time x virus interaction.  F_(29, 609)_ = 6.51, *p* <0.0001 |  |
|  | F |  | t_(21)_= 4.52, *p* = 0.0002 | SST = 32.41 ± 5.221  PV = 4.388 ± 3.676 |
| 6 | B | vehicle, n = 13 slices of 4 female mice  Dyn, n = 12 slices of 4 female mice | t_(23)_= 1.76, *p* = 0.0903 | Vehicle = 1.232 ± 0.0938  Dyn = 0.9819 ± 0.1064 |
|  | C | vehicle, n = 16 slices of 4 male and 2 female mice  Dyn, n = 16 slices of 4 male and 2 female mice | t_(30)_= 4.00, *p* = 0.0004 | Vehicle = 0.8821 ± 0.0571  Dyn = 2.306 ± 0.3510 |
|  | E | BLA, vehicle = 11 cells and Dyn = 8 cells in 1 male and 3 female mice  VH, vehicle = 5 cells and Dyn = 9 cells in 2 male and 2 female mice |  |  |
|  | F |  | One-way ANOVA.  F_(2, 20)_ = 12.78, *p* = 0.0003; **p* = 0.0417, ***p* = 0.0076 | Vehicle = 0.9232 ± 0.0741  VH = 1.442 ± 0.1334  BLA = 0.5687 ± 0.1365 |
|  | G | n = 7 cells in 1 male and 3 female mice | t_(12)_ = 0.09713, *p* = 0.9242 | BL+PTX = 1.000 ± 0.000 Dyn+PTX = 1.004 ± 0.042 |
|  | H | BLA, vehicle = 7 cells and Dyn = 11 cells in 1 male and 4 female mice  VH, vehicle = 4 cells and Dyn = 8 cells in 2 male and 2 female mice |  |  |
|  | I |  | One-way ANOVA.  F_(2, 24)_ = 29.57, *p* < 0.0001;  *****p* < 0.0001 | Vehicle = 1.006 ± 0.0522  VH = 2.304 ± 0.2917  BLA = 0.5521 ± 0.1338 |
|  | J | n = 4 cells in 1 male and 3 female mice | t_(6)_ = 0.4332, *p* = 0.68 | BL+PTX = 1.000 ± 0.000 Dyn+PTX = 0.914 ± 0.199 |
|  | L | n = 10 cells in 1 male and 2 female mice | Paired t-test.  t_(18)_= 2.248, *p* = 0.0374 | VH = 1.507 ± 0.221  BLA = 0.8411 ± 0.197 |
| S6 | B | Dyn = 12 cells and Dyn+nor-BNI = 7 cells in 4 male and 3 female mice | t_(17)_= 2.339, *p* = 0.0318 |  |
|  | D | VH = 45 cells and BLA+VH = 45 cells in 3 male and 7 female mice | t_(82)_= 5.288, *p* < 0.0001 |  |

**Table 2. Table containing information about all the statistical analysis conducted throughout the project.**
